# Meta-coexpression conservation analysis of microarray data: a "subset" approach provides insight into brain-derived neurotrophic factor regulation

**DOI:** 10.1186/1471-2164-10-420

**Published:** 2009-09-08

**Authors:** Tamara Aid-Pavlidis, Pavlos Pavlidis, Tõnis Timmusk

**Affiliations:** 1 Department of Gene Technology, Tallinn University of Technology, Akadeemia tee 15, 19086 Tallinn, Estonia; 2 Department of Biology, Section of Evolutionary Biology, University of Munich, Grosshaderner Strasse 2, 82152 Planegg-Martinsried, Germany

## Abstract

**Background:**

Alterations in brain-derived neurotrophic factor (*BDNF*) gene expression contribute to serious pathologies such as depression, epilepsy, cancer, Alzheimer's, Huntington and Parkinson's disease. Therefore, exploring the mechanisms of *BDNF *regulation represents a great clinical importance. Studying *BDNF *expression remains difficult due to its multiple neural activity-dependent and tissue-specific promoters. Thus, microarray data could provide insight into the regulation of this complex gene. Conventional microarray co-expression analysis is usually carried out by merging the datasets or by confirming the re-occurrence of significant correlations across datasets. However, co-expression patterns can be different under various conditions that are represented by subsets in a dataset. Therefore, assessing co-expression by measuring correlation coefficient across merged samples of a dataset or by merging datasets might not capture all correlation patterns.

**Results:**

In our study, we performed meta-coexpression analysis of publicly available microarray data using *BDNF *as a "guide-gene" introducing a "subset" approach. The key steps of the analysis included: dividing datasets into subsets with biologically meaningful sample content (e.g. tissue, gender or disease state subsets); analyzing co-expression with the *BDNF *gene in each subset separately; and confirming co- expression links across subsets. Finally, we analyzed conservation in co-expression with *BDNF *between human, mouse and rat, and sought for conserved over-represented TFBSs in *BDNF *and BDNF-correlated genes. Correlated genes discovered in this study regulate nervous system development, and are associated with various types of cancer and neurological disorders. Also, several transcription factor identified here have been reported to regulate *BDNF *expression *in vitro *and *in vivo*.

**Conclusion:**

The study demonstrates the potential of the "subset" approach in co-expression conservation analysis for studying the regulation of single genes and proposes novel regulators of *BDNF *gene expression.

## Background

The accumulation of genome-wide gene expression data has enabled biologists to investigate gene regulatory mechanisms using system biology approaches. Recent developments in microarray technologies and bioinformatics have driven the progress of this field [1]. Moreover, publicly available microarray data provide information on human genome-wide gene expression under various experimental conditions, which for most researchers would be difficult to access otherwise.

BDNF (brain-derived neurotrophic factor) plays an important role in the development of the vertebrates' nervous system [2]. BDNF supports survival and differentiation of embryonic neurons and controls various neural processes in adulthood, including memory and learning [3], depression [4], and drug addiction [5]. Alterations in *BDNF *expression can contribute to serious pathologies such as epilepsy, Huntington, Alzheimer's, and Parkinson's disease [6]. Alteration in *BDNF *expression is associated with unfavorable prognosis in neuroblastoma [7], myeloma [8], hepatocellular carcinoma [9] and other tumors [10]. Apart from brain, expression of alternative *BDNF *transcripts has been detected in a variety of tissues (such as heart, muscle, testis, thymus, lung, etc.) [11,12]. Numerous studies have been conducted to unravel the regulation of *BDNF *expression in rodents and human. Data on the structure of human [11] and rodent [12]*BDNF *gene have been recently updated. Nevertheless, little is known about the regulation of human BDNF gene expression *in vivo*. Unraveling the regulation of *BDNF *expression remains difficult due to its multiple activity-dependent and tissue-specific promoters. Thus, analysis of the gene expression under various experimental conditions using microarray data could provide insight into the regulation of this complex gene.

Meta-coexpression analysis uses multiple experiments to identify more reliable sets of genes than would be found using a single data set. The rationale behind meta-coexpression analysis is that co-regulated genes should display similar expression patterns across various conditions. Moreover, such analysis may benefit from a vast representation of tissues and conditions [13]. A yeast study showed that the ability to correctly identify co-regulated genes in co-expression analysis is strongly dependent on the number of microarray experiments used [14]. Another study that examined 60 human microarray datasets for co-expressed gene pairs reports that gene ontology (GO) score for gene pairs increases steadily with the number of confirmed links compared to the pairs confirmed by only a single dataset [15]. Several studies have successfully applied meta-analysis approach to get important insights into various biological processes. For instance, microarray meta-analysis of aging and cellular senescence led to the observation that the expression pattern of cellular senescence was similar to that of aging in mice, but not in humans [16]. Data from a variety of laboratories was integrated to identify a common host transcriptional response to pathogens [17]. Also, meta-coexpression studies have displayed their efficiency to predict functional relationships between genes [18]. However, co-expression alone does not necessarily imply that genes are co-regulated. Thus, analysis of evolutionary conservation of co-expression coupled with the search for over-represented motifs in the promoters of co-expressed genes is a powerful criterion to identify genes that are co-regulated from a set of co-expressed genes [19,20].

In co-expression analysis, similarity of gene expression profiles is measured using correlation coefficients (CC) or other distance measures. If the correlation between two genes is above a given threshold, then the genes can be considered as «co-expressed» [1]. Co-expression analysis using a «guide-gene» approach involves measuring CC between pre-selected gene(s) and the rest of the genes in a dataset.

It is a common practice in meta-coexpression studies to assess co-expression by calculating the gene pair correlations after merging the datasets [20] or by confirming the re-occurrence of significant correlations across datasets [15]. However, it has been shown recently that genes can reveal differential co-expression patterns across subsets in the same dataset (e.g. gene pairs that are correlated in normal tissue might not be correlated in cancerous tissue or might be even anti-correlated) [21]. Therefore, assessing co-expression by measuring CC across merged samples of a dataset or by merging datasets may create correlation patterns that could not be captured using the CC measurement.

In this study, we performed co-expression analysis of publicly available microarray data using *BDNF *as a "guide-gene". We inferred *BDNF *gene co-expression links that were conserved between human and rodents using a novel "subset" approach. Then, we discovered new putative regulatory elements in human *BDNF *and in BDNF-correlated genes, and proposed potential regulators of *BDNF *gene expression.

## Results

**Figure 1:**
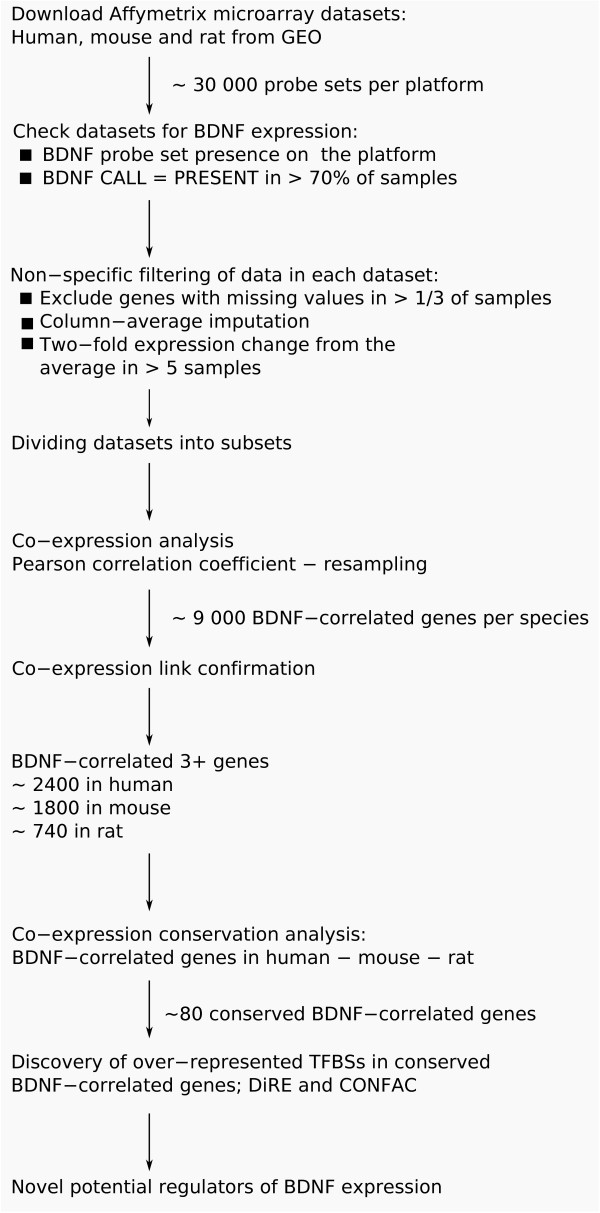
**Microarray data analysis flowchart**. Altogether, 80 human, mouse and rat Affymetrix datasets were analyzed (dataset selection criteria: > 16 samples per dataset; BDNF detection call PRESENT in more than 70% of the samples). Data was subjected to non-specific filtering (missing values and 2-fold change filtering). Thereafter, datasets were divided into 299 corresponding subsets. Co-expression analysis in human, mouse and rat subsets allowed the detection of genes that co-expressed with *BDNF *in more than 3 subsets (~1000 genes for each species). As a result of co-expression conservation analysis, 84 genes were found to be correlated with *BDNF *in all three species. Discovery of over-represented motifs in the regulatory regions of these genes and in *BDNF *suggested novel regulators of *BDNF *gene expression.

We analyzed 299 subsets derived from the total of 80 human, mouse and rat microarray datasets. In order to avoid spurious results that could arise from high-throughput microarray analysis methods, we applied successive filtering of genes. Then, we divided datasets into subsets with biologically meaningful sample content (e.g. tissue, gender or disease state subsets), analyzed co-expression with *BDNF *across samples separately in each subset and confirmed the links across subsets. Finally, we analyzed conservation in co-expression between human, mouse and rat, and sought for conserved TFBSs in *BDNF *and BDNF-correlated genes (Figure 1).

### Data filtering

Gene Expression Omnibus (GEO) from NCBI and ArrayExpress from EBI are the largest public peer reviewed microarray repositories, each containing about 8000 experiments. In order to avoid inaccuracies arising from measuring expression correlation across different microarray platforms [13] we used only Affymetrix GeneChips platforms for the analysis. Since ArrayExpress imports Affymetrix experiments from GEO , we used only GEO database to retrieve datasets.

A study examining the relationship between the number of analyzed microarray experiments and the reliability of the results reported that the accuracy of the analysis plateaus at between 50 and 100 experiments [14]. Another study demonstrated how the large amount of microarray data can be exploited to increase the reliability of inferences about gene functions. Links that were confirmed three or more times between different experiments had significantly higher GO term overlaps than those seen only once or twice (*p *< 10^-15^) [15]. Therefore, we performed meta-coexpression analysis using multiple experiments to increases the accuracy of the prediction of the co-expression links.

Since *BDNF *served as a guide-gene for our microarray study, qualitative and quantitative criteria were applied for selection of the experiments with respect to *BDNF *probe set presence on the platform [see Additional file 1: BDNF probe sets], *BDNF *signal quality and expression levels. In addition, non-specific filtering [19] was performed to eliminate the noise (see Methods/Microarray datasets). Consequently, 80 human, mouse and rat microarray experiments (datasets) from Gene Expression Omnibus (GEO) database met the selection criteria. Each dataset was split into subsets according to the annotation file included in the experiment [see Additional file 2: Microarray datasets and Additional file 3: Subsets]. In summary, 299 subsets were obtained from 38 human, 24 mouse and 18 rat datasets. From 38 human datasets, 8 were related to neurological diseases (epilepsy, Huntington's, Alzheimer's, aging, encephalitis, glioma and schizophrenia) and contained samples from human brain; another 9 datasets contained samples from human "normal" (non-diseased) tissues (non-neural, such as blood, skin, lung, and human brain tissues); 12 datasets had samples from cancerous tissues of various origins (lung, prostate, kidney, breast and ovarian cancer). The rest 9 datasets contained samples from diseased non-neural tissues (HIV infection, smoking, stress, UV radiation etc.). Out of 24 mouse datasets, 5 datasets were related to neurological diseases (brain trauma, spinal cord injury, amyotrophic lateral sclerosis, and aging); 15 datasets contained normal tissue samples (neural and peripheral tissues); 1 dataset contained lung cancer samples; 3 datasets were related to non-neural tissues' diseases (muscle dystrophy, cardiac hypertrophy and asthma). Among 18 rat datasets, 11 datasets were related to neurological diseases (spinal cord injury, addiction, epilepsy, aging, ischemia etc), 5 datasets were with "normal tissue samples" composition and 2 datasets examined heart diseases [see Additional file 2: Microarray datasets].

According to Elo and colleagues [22] the reproducibility of the analysis of eight samples approaches 55%. Selecting subsets with more than eight samples for the analysis could increase the reproducibility of the experiment however reducing the coverage, since subsets with lower number of samples would be excluded. Thus, we selected subsets with a minimum of eight samples for the analysis, in order to achieve satisfactory reproducibility and coverage. The expression information for human, mouse and rat genes obtained from GEO database, information about *BDNF *probe names used for each dataset, information about subsets derived from each experiment, and data on correlation of expression between BDNF and other genes for each microarray subset has been made available online and can be accessed using the following link: .

### Differential expression of BDNF across subsets

Since the study was based on analyzing subsets defined by experimental conditions (gender, age, disease state etc) it was of biological interest to examine if *BDNF *is differentially expressed across subsets within a dataset. We used Kruskal-Wallis test [23] to measure differential expression. The results of this analysis are given in the Additional files 4, 5 and 6: Differential expression of the BDNF gene in human, mouse and rat datasets.

### Co-expression analysis

Since the expression of *BDNF *alternative transcripts is tissue-specific and responds to the variety of stimuli, seeking for correlated genes in each subset separately could help to reveal condition-specific co-expression. The term "subset" in this case must be understood as "a set of samples under the same condition".

We derived 119 human, 73 mouse and 107 rat subsets from the corresponding datasets. Pearson correlation coefficient (PCC) was chosen as a similarity measure since it is one of the most commonly used, with many publications describing analysis of Affymetrix platforms [13,24,25]. PCC between *BDNF *and other genes' probe sets was measured across samples for each subset separately. From each subset, probe sets with PCC r > 0.6 were selected. It was demonstrated by Elo and colleagues [22] that in the analysis of simulated datasets a cutoff value r = 0.6 showed both high reproducibility (~0.6 for profile length equal to 10) and low error. A "data-driven cutoff value" approach has been rejected because it is based on the connectivity of the whole network, whereas we focused only on the links between *BDNF *and other genes. A lower threshold of 0.4 generated a list of genes that showed no significant similarities when analyzed using g:Profiler tool that retrieves most significant GO terms, KEGG and REACTOME pathways, and TRANSFAC motifs for a user-specified group of genes [26]. The value r = 0.6 was chosen over more stringent PCC values because the lengths of the expression profiles were not too short (mean profile length ~17, standard deviation ~12). Moreover, the PCC threshold higher than 0.6 was not justified since we performed further filtering by selecting only conserved correlated genes, thus controlling the spurious results.

Each probe set correlation with *BDNF *that passed the threshold was defined as a "link". It has been previously shown that a link must be confirmed in at least 3 experiments (3+ link) in order to be called reliable [15]. Therefore, we selected (3+) genes for evolutionary conservation analysis, narrowing the list of correlated genes to eliminate the noise. g:Profiler analysis of these genes revealed that the results are statistically significant (low p-values) and the genes belong to GO categories that are relevant to biological functions of BDNF. For example, the list of human genes produced the following results when analyzed with g:Profiler (*p*-values for the GO categories are given in the parenthess): nervous system development (5.96·10^-21^), central nervous system development (3.29·10^-07^), synaptic transmission (4.40·10^-11^), generation of neurons (1.58·10^-08^), neuron differentiation (1.02·10^-06^), neurite development (4.11·10^-07^), heart development (1.67·10^-09^), blood vessel development (5.51·10^-14^), regulation of angiogenesis (7.16·10^-09^), response to wounding (1.32·10^-11^), muscle development (1.53·10^-10^), regulation of apoptosis (1.65·10^-07^), etc.

We have used r = 0.6 as a "hard" threshold value for the CC. A disadvantage of this approach is that there will be no connection between *BDNF *and other genes whose correlation with *BDNF *is 0.59 in a specific dataset [27]. Using multiple datasets was expected to remedy this effect. An alternative approach would be to use "soft" threshold approaches [27]. According to the soft threshold approach, a weight between 0 and 1 is assigned to the connection between each pair of genes (or nodes in a graph). Often, the weight between the nodes *A *and *B *is represented by some power of the CC between *A *and *B*. However, other similarity measures may be used given that they are restricted in [0, 1]. A drawback of the weighted CC approach is that it is not clear how to define nodes that are directly linked to a specific node [27] because the available information is related only to how strongly two nodes are connected. Thus, if neighbors to a node are requested, threshold should be applied to the connection strengths. Alternatively, Li and Horvath [28] have developed an approach to answer this question based on extending the topological overlap measure (TOM), which means that the nodes (e.g. genes) should be strongly connected and belong to the same group of nodes. However, this analysis requires the whole network of a set of genes. In the current analysis, we did not construct the co-expression network for all the genes of microarray experiments. Instead, we focused on a small part of it i.e. the *BDNF *gene and the genes linked to *BDNF*. Therefore, TOM analysis was not possible using our approach.

To see how the "weighted CC" method would affect the results of our study we used a simplified approach. Instead of applying "hard" threshold (0.6) for the CC we measured the strength of all the connections between *BDNF *and all the genes in a microarray experiment. The connection strength s_j _= [(1 + CC_j_)/2]^b^, where CC_j _denotes the CC between *BDNF *and the gene j, is between 0 and 1 and b is an integer. In order to define b, analysis of the scale-free properties of the network is required. However, we used the value 6. Great b values give lower weight to weak connections. Then we calculated the average s_j_(ave(s_j_)) among all the subsets. Finally, we sorted the genes based on their ave(s_j_) and calculated the overlap of the top of this list with our results for each species (human mouse and rat). When restricting the top of the weighted CC list to the same number of genes that we have obtained for the 3+ list for each species, we observed that the top-weighted CC genes overlap extensively with the 3+ list (overlapping > 80%) for each species. Therefore, even though the "soft" and "hard" thresholding approaches are considerably different we observe quite extensive overlap of the results. We would like to stress that we did not apply the full weighted CC and TOM methodology since it would require the construction of the whole network which was beyond the aims of our study. However, such investigation of the whole co-expression network could contribute to the understanding of *BDNF *regulation and function.

### Correlation conservation and g:Profiler analysis

Co-expression that is conserved between phylogenetically distant species may reveal functional gene associations [29]. We searched for common genes in the lists of 2436 human, 1824 mouse and 740 rat genes (3+ genes, whose expression is correlated with *BDNF*). From these genes, 490 were found to be correlated with *BDNF *in human and mouse, 210 correlated with *BDNF *in human and rat, and 207 conserved between mouse and rat [see Additional file 7: Conserved BDNF-correlated genes]. We found a total of 84 genes whose co-expression with *BDNF *was conserved in all three organisms (Table 1) [see also Additional file 7: Conserved BDNF-correlated genes].

**Table 1:** BDNF-correlated genes conserved between human, mouse and rat.

**GO category**	**Conserved correlated genes**											
protein tyrosine kinase PW *	ANGPT1	BAIAP2	DUSP1	EPHA4	EPHA5	EPHA7	FGFR1	GAS6	KALRN	IRS2	NTRK2	
	PTPRF	FP106										

dendrite localization*	DBN1	FREQ	GRIA3	KCND2	NTRK2							

signal transduction*	ANGPT1	CREM	DUSP6	EPHA5	FGFR1	IGFBP5	KALRN	NR4A2	PDE4B	PRKAG2	PTPRF	TBX3
	BAIAP2	CXCL5	EGR1	EPHA7	GAS6	IL6ST	KLF10	NTRK2	PENK	PRKCB	RGS4	ZFP106
	COL11A1	DUSP1	EPHA4	FGF13	GRIA3	IRS2	MYH9	ODZ2	PLAUR	PRKCE	SCG2	

hsa-miR-369-3p*	COL11A1	DBC1	DCN	DUSP1	GAS6	ITF-2	KLF10	NEUROD6	PENK	TRPC4		

TF: CCCGCCCCCRCCCC (KROX) *	ATF3	ATP1B1	CCND2	COL11A1	DBN1	DLGAP4	EPHA7	GAS6	GRIA3	IL6ST	IRS2 KCND2	
	KLF10	NFIA	NPTXR	PCSK2	SNCA	THRA						

TF: GGGGAGGG (MAZ/SP1) *	ATF3	CCND2	DBC1	DUSP6	FREQ	ITF-2	MBP	NPTXR	PCSK1	PTGS2	THRA	
	BAIAP2	COL4A5	DBN1	EGR1	GRIA3	KALRN	MDM2	NR4A2	PDE4B	PTPRF	TRPC4	
	BASP1	CREM	DLGAP4	EPHA5	HN1	KLF10	NFIA	NTRK2	PRKCB1	PURA	VCAN	
	CAMK2D	CXCL5	DUSP1	EPHA7	IRS2	LMO7	NPTX1	OLFM1	PRSS23	TBX3		

NS development*	BAIAP2	EPHA4	FGF13	IRS2	MBP	NEUROD6	NR4A2	OLFM1	PTPRF	SMARCA4	TBX3	
	DBN1	EPHA7	FGFR1	KALRN	NEFL	NPTX1	NTRK2	PCSK2	PURA	SNCA		

angiogenesis	ANGPT1	BAIAP2	CYR61	MYH9	SCG2	SERPINE1	TBX3					

apoptosis/anti-apoptosis	BIRC4	KLF10	NEFL	PLAGL1	PRKCE	SCG2	SNCA	TBX3				

cell cycle	CAMK2D	CORO1A	DUSP1	MDM2	MYH9	PPP3CA						

synaptic transmission/plasticity	DBN1	KCND2	MBP	NPTX1	NR4A2	SNCA						

GO categories marked with a star (*) have been reported as statistically significant for this gene list by g:Profiler analysis tool. Human gene names are given representing mouse and rat orthologs whenever gene names for all three species are not the same. GO - gene ontology, PW - pathway, TF - transcription factor, NS - nervous system.

Due to a variety of reasons (e.g. sample size of a dataset/subset, probe set binding characteristics, sample preparation methods, etc.), when measured only in one dataset/subset, some of the co-expression links might occur by chance. Checking for multiple re-occurrence of a link is expected to reduce the number of false-positive links. More importantly, the conservation analysis should further reduce the number of artifacts. However, since our analysis comprised a multitude of subsets it was important to estimate the statistical significance of the results. To tackle this problem, we created randomized subsets similarly to what was described by Lee and colleagues [15] and calculated the distribution of correlated 3+ links for each species separately. The results showed that our co-expression link confirmation analysis resulted in a significantly higher number of links compared to the randomized data (*p*-value < 0.005 for each species). However, it should be mentioned that the number of 3+ links remained quite high in the randomized datasets: for human subsets it constituted about 58% of the observed 3+ links, for mouse about 43% and for rat 21%. These results justify the subsequent co-expression conservation analysis step. Indeed, in random human, mouse and rat subsets the number of correlated 3+ links was only about 9% of the discovered conserved BDNF-correlated links (that is ~7.5 genes out of 84).

Analysis of the list of 84 conserved BDNF-correlated genes using g:Profiler showed significantly low *p*-values for all the genes and revealed significant GO categories related to BDNF actions [see Additional file 8: g:Profiler analysis]. Statistically significant GO categories included: i) MYC-associated zinc finger protein (MAZ) targets (44 genes, *p *= 1.82·10^-05^); ii) signal transduction (36 genes, *p *= 3.51·10^-06^); iii) nervous system development (17 genes, *p *= 5.27·10^-08^); iv) Kruppel-box protein homolog (KROX) targets (18 genes, *p *= 1.21·10^-04^); v) transmembrane receptor protein tyrosine kinase pathway (7 genes, *p *= 3.56·10^-06^); vi) dendrite localization (5 genes, *p *= 1.82·10^-05^) (Table 1).

According to the Gene Ontology database, conserved BDNF-correlated gene products participate in axonogenesis (*BAIAP2*), dendrite development (*DBN1*), synaptic plasticity and synaptic transmission (*DBN1*, *KCND2*, *MBP*, *NPTX1*, *NR4A2 *and *SNCA*), regeneration (*GAS6*, *PLAUR*), regulation of apoptosis (*XIAP *(known as *BIRC4*), *KLF10*, *NEFL, PLAGL1, PRKCE, SCG2, SNCA*, and *TBX3*), skeletal muscle development (*MYH9, PPP3CA*, and *TBX3*) and angiogenesis (*ANGPT1, BAIAP2, CYR61, MYH9, SCG2, SERPINE1 *and *TBX3*) (Table 1). Out of 84, 24 BDNF-correlated genes are related to cancer and 14 are involved in neurological disorders (Table 2).

**Table 2:** Conserved correlated genes are associated with various types of cancer and neurological disorders.

**Disease**	**Associated genes**	**References**
Schizophrenia	BDNF RGS4 NR4A2	Schmidt-Kastner et al. (2006)

Parkinson's disease	BDNF PTGS2 SNCA NR4A2	Murer et al. (2001) Chae et al. (2008) Pardo and van Duijn (2005)

Alzheimer's	BDNF KALRN	Murer et al. (2001) Youn et al. (2007)

Polyglutamine neurodegeneration	NEFL BAIAP2	Mosaheb et al. (2005) Thomas et al. (2001)

alpha-mannosidosis	MAN1A1	D'Hooge et al. (2005)

Ophthalmopathy	CYR61 DUSP1 EGR1 PTGS2	Lantz et al. (2005)

Epilepsy	BDNF DUSP6 EGR1	Binder and Scharfman (2004) Rakhade et al. (2007)

Depression	BDNF DUSP1	Russo-Neustadt and Chen (2005) Rakhade et al. (2007)

Ischemia	BDNF CD44 PTGS2	Binder and Scharfman (2004) Murphy et al. (2005)

Ovarian carcinoma	BDNF ITF2 DUSP1 RGS4	Yu et al. (2008) Kolligs et al. (2002) Puiffe et al. (2007)

Breast cancer	BDNF FGFR1 CCND2 PLAU SERPINE1 PLAUR MAZ DUSP6 EGR1 KFL10 PTRF	Tozlu et al. (2006) Koziczak et al. (2004) Grebenchtchikov et al. (2005) Cui et al. (2006) Liu et al. (2007) Reinholz et al. (2004) Levea et al. (2000)

Lung cancer	BDNF ODZ2 CCND2 GFI1	Ricci et al. (2005) Kan et al. (2006)

Prostate cancer	BDNF IGFBP5 PLAUR p75NTR	Bronzetti et al. (2008) Nalbandian et al. (2005)

Pheochromocytoma	PCSK1 PCSK2 SCG2	Guillemot et al. (2006)

Endometrial cancer	CXCL5 OLFM1	Wong et al. (2007)

Leukemia	PKCB1 CCND2	Hans et al. (2005)

### Interactions among correlated genes

**Figure 2:**
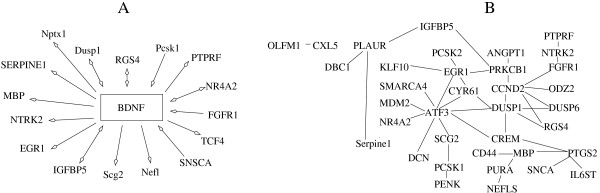
**Reported interactions between conserved correlated genes in human**. Connections between the genes were created by accessing the literature using iHOP tool. (A) Interactions between correlated genes and *BDNF*. Arrows: "↔" co-expression or co-regulation; "BDNF← "regulation of *BDNF*; "BDNF→" regulation by BDNF. (B) Connections among correlated genes.

We searched if any of the correlated genes had known interactions with BDNF using Information Hyperlinked over Proteins gene network (iHOP). iHOP allows navigating the literature cited in PubMed and gives as an output all sentences that connect gene A and gene B with a verb [30]. We constructed a "gene network" using the iHOP Gene Model tool to verify *BDNF*-co-expression links with the experimental evidences reported in the literature (Figure 2). For the URL links to the cited literature see Additional file 9: iHOP references.

According to the literature, 17 out of 84 conserved correlated genes have been reported to have functional interaction or co-regulation with BDNF (Figure 2A). *IGFBP5 *[31], *NR4A2, RGS4 *[32] and *DUSP1 *[33] have been previously reported to be co-expressed with human or rodent *BDNF*. Other gene products, such as FGFR1 [34] and SNCA [35] are known to regulate *BDNF *expression. Proprotein convertase PCSK1 is implied in processing of pro-BDNF [36]. PTPRF tyrosine phosphatase receptor associates with NTRK2 and modulates neurotrophic signaling pathways [37]. Thyroid hormone receptor alpha (THRA) induces expression of BDNF receptor NTRK2 [38]. Finally, expression of such genes like *EGR1 *[39], *MBP *[40], *NEFL *[41], *NPTX1 *[42], *NTRK2*, *SERPINE1 *[43], *SCG2 *[44], *SNCA *[45] and *TCF4 *(also known as *ITF2*) [46] is known to be regulated by BDNF signaling. *CCND2*, *DUSP1*, *DUSP6*, *EGR1 *and *RGS4 *gene expression is altered in cortical GABA neurons in the absence of BDNF [47].

iHOP reports the total of 250 interactions with human BDNF. In order to assess the probability of observing 17/84 or more functional interactions between BDNF and other genes, we had to make an assumption regarding the total number of human genes that iHOP uses. A lower number of total genes would result in higher *p*-values whereas a higher number of total genes would produce lower *p*-values. We assumed that the total number of human genes is N = 5000, 10000, 20000 or 30000. Furthermore, the total number of genes linked to BDNF is m = 250 based on iHOP data. Thus, the *p*-values were obtained using the right-tail of the hypergeometric probability distribution. For N = 5000, 10000, 20000 or 30000, the *p*-values are 1.0 × 10^-07^, 1.7 × 10^-12^, 1.3 × 10^-17^, 1.18 × 10^-20 ^respectively.

By analyzing the iHOP network indirect connections with BDNF could be established for the genes that did not have known direct interactions with BDNF (Figure 2B). For example, SCG2 protein is found in neuroendocrine vesicles and is cleaved by PCSK1 [48] - protease that cleaves pro-BDNF. BDNF and NTRK2 signaling affect *SNCA *gene expression and alpha-synuclein deposition in substantia nigra [49]. *ATF3 *gene is regulated by EGR1 [50], which expression is activated by BDNF [39]. For more interactions see Figure 2.

### Motif discovery

Assuming that genes with similar tissue-specific expression patterns are likely to share common regulatory elements, we clustered co-expressed genes according to their tissue-specific expression using information provided by TiProd database [51]. Each tissue was assigned a category and the genes expressed in corresponding tissues were clustered into the following categories: i) CNS, ii) peripheral NS (PNS), ii) endocrine, iii) gastrointestinal, and iv) genitourinary. We applied DiRE [52] and CONFAC [53] motif-discovery tools to search for statistically over-represented TFBSs in the clusters and among all conserved BDNF-correlated genes. DiRE can detect regulatory elements outside of proximal promoter regions, as it takes advantage of the full gene locus to conduct the search. The software predicts function-specific regulatory elements (REs) consisting of clusters of specifically associated and conserved TFBSs, and it also scores the association of individual TFs with the biological function shared by the group of input genes [52]. DiRE selects a set of candidate REs from the gene loci based on the inter-species conservation pattern which is available in the form of precomputed alignments of genomic sequence from fish, rodent, human and other vertebrate lineages [54]. This type of the alignment enables the tool to detect regulatory elements that are phylogenetically conserved at the same genomic positions in different species. CONFAC software [53] enables the identification of conserved enriched TFBSs in the regulatory regions of sets of genes. To perform the search, human and mouse genomic sequences from orthologous gene pairs are compared by pairwise BLAST, and only significantly conserved (e-value < 0.001) regions are analyzed for TFBSs.

**Figure 3:**
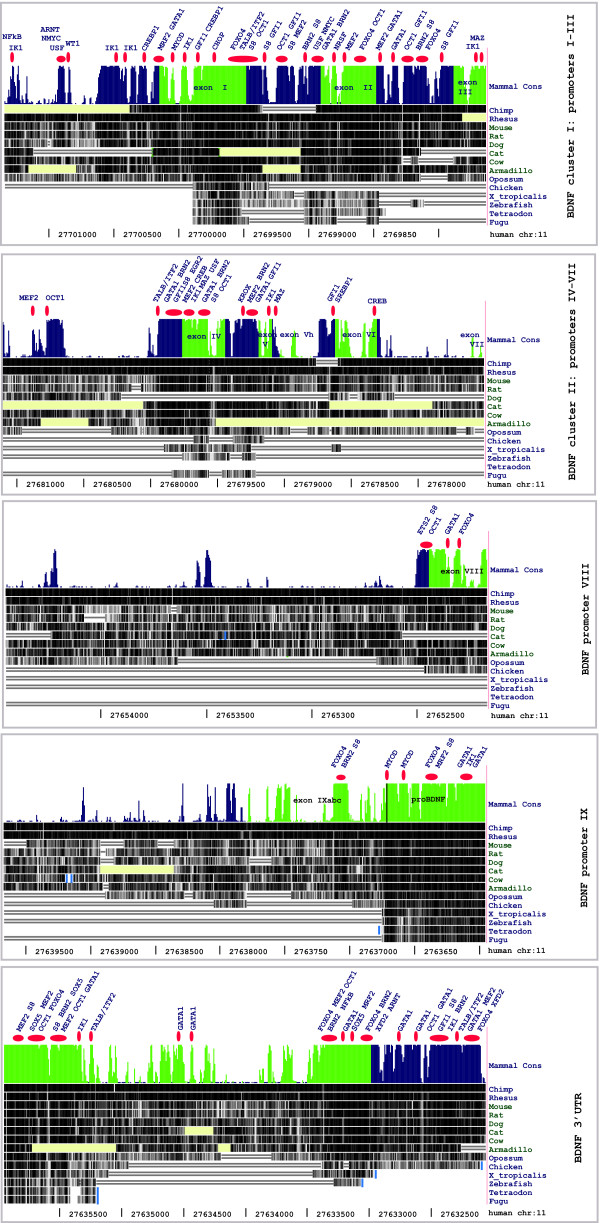
**Novel regulatory elements in the BDNF gene**. Highly conserved TFBSs in the *BDNF *locus as predicted by DiRE and CONFAC tools. Given TFBSs were also found to be over-represented in the BDNF-correlated genes. Histograms represent evolutionary conservation across 9 mammal species (adapted from UCSC Genome Browser at ) (39). The height of the histogram reflects the size of the conservation score. Conservation for each species is shown in grayscale using darker values to indicate higher levels of overall conservation. Missing sequences are highlighted by regions of yellow. Single line: no bases in the aligned species; double line: aligning species has one or more unalignable bases in the gap region. Transcribed regions (*BDNF *exons and 3'UTR) are highlighted in green; non-transcribed regions (*BDNF *promoters and introns) are highlighted in blue. Red ovals represent TFBSs mapped to the *BDNF *gene sequences. Mapped TFBSs have Matrix Similarity score >0.85 and Core Similarity score >0.99. Core elements of presented TFBSs have 100% of conservation across mammals. For the structure of human *BDNF *see Pruunsild et al., 2007 [11].

Using DiRE we discovered two regulatory regions at the human *BDNF *locus that were enriched in TFBSs (Figure 3) [see also Additional file 10: DiRE motif discovery results for BDNF and 84 conserved correlated genes]. The first regulatory region spans 218 bp and is located 622 bp upstream of human *BDNF *exon I transcription start site (TSS). The second putative regulatory region is 1625 bp long and located 2915 bp downstream of the *BDNF *stop-codon. Analysis of mouse and rat gene lists produced similar results. Significant over-representation of binding sites for WT1, KROX, ZNF219, NFkB, SOX, CREB, OCT, MYOD and MEF2 transcription factors was reported by DiRE in *BDNF *and BDNF-correlated genes when all the genes were analyzed as one cluster [see Additional file 10: DiRE motif discovery results for BDNF and 84 conserved correlated genes]. Also, the following cluster-specific over-representation of TFBSs was detected: i) CNS - KROX; ii) endocrine - TAL1beta/TCF4, ETS2, SOX5, and ARID5B (known as MRF2); iii) gastrointestinal - MMEF2, and SREBF1; iv) genitourinary - ATF4/CREB, and GTF3 (TFIII) (Table 3) [see also Additional file 11: DiRE motif discovery results for conserved BDNF-correlated genes clustered by tissue-specific expression].

**Table 3:** Over-represented conserved TFBSs in human BDNF and in the BDNF-correlated genes as predicted by DiRE and CONFAC.

**TFBS**	***p*-value** **CONFAC**	**Target genes**
ARNT	0.012	*BDNF pI-II, BDNF 3'UTR; PRKCE, USP2, CAMK2D, CCND2, NEUROD6, THRA, DUSP1, CBX6, ATP1B1, FREQ, ITF-2*

POU3F2 (BRN2)	< 0.001	*BDNF pII-V, BDNF exon II, IV, IX, BDNF3'UTR; USP2, CAMK2D, THRA, NFIA, PRSS23, CBX6, CUGBP2, EPHA5, EPHA7, BAIAP2, RKCE, CPD, EPHA4, IL6ST, CCND2, DUSP6, KCND2, MAN1A1, SCG2, GRIA3, COL11A1, TRPC4, FGF13, HN1, ANGPT1, TCF4, MYH9, PCSK1*

CHOP	NA	*BDNF I, COL11A1, CD44, BAIAP2, PPP3CA, IL6ST, NEUROD6, SCG2, CYR61, IGFBP5, THRA, NFIA, FGF13, ATP1A2, ANGPT1, DBC1, CUGBP2, EGR1*

CREB	0.013	*BDNF pI, IV, VI, BDNF exon I; BAIAP2, PRKCE, USP2, EPHA4, CAMK2D, CCND2, FGFR1, CYR61, GRIA3, THRA, DUSP1, PENK, PCSK1, PCSK2, HN1, ATP1B1, EGR1, COL4A5, KLF10, EPHA4, FGF13, CBX6, CUGBP2, EPHA5*

ETS2	NA	*BDNF pII, VIII; THRA, EPHA7, FGF13, BAIAP2 and NFIA promoters, and in COL11A1, PLAGL1, and XIAP intergenic regions*

FOXO4	< 0.001	*BDNF exon I, II, VIII, IX, BDNF pIII, IV, BDNF 3'UTR; CD44, TBX3, BAIAP2, PPP3CA, CPD, USP2, PRKCB, EPHA4, CORO1A, CAMK2D, NEUROD6, FGFR1, SCG2, CYR61, GRIA3, THRA, NFIA, COL11A1, DUSP1, TRPC4, PRSS23, PCSK2, ANGPT1, FREQ, PRKAG2, TCF4, MYH9, PCSK1, DBC1, CUGBP2, EGR1, EPHA5*

GATA1	< 0.001	*BDNF pI, III-V, BDNF exon I, II, VIII, IX, BDNF 3'UTR; CD44, TBX3, SNCA, PPP3CA, PRKCE, COL4A5, USP2, EPHA4, IL6ST, SLC4A7, CAMK2D, ATF3, CCND2, NEUROD6, DUSP6, KCND2, SCG2, CYR61, IGFBP5, THRA, NFIA, COL11A1, PENK, FGF13, PRSS23, ATP1B1, ATP1A2, ANGPT1, DBC1, CUGBP2, EGR1*

GFI1	< 0.001	*BDNF exon I, BDNF pII-VI, BDNF 3'UTR; SNCA, ATP1A2, MYH9, DBC1, CD44, BAIAP2, PPP3CA, PRKCE, COL4A5, CPD, USP2, EPHA4, IL6ST, SLC4A7, CAMK2D, CCND2, NEUROD6, KCND2, SCG2, CYR61, IGFBP5, THRA, NFIA, COL11A1, DUSP1, TRPC4, PENK, FGF13, PRSS23, PCSK2, ATP1B1, PTPRF, ANGPT1, TCF4, CUGBP2, EGR1, EPHA5, EPHA7*

IK1 (ikaros)	< 0.001	*BDNF pI, BDNF exon I-V, IX, BDNF 3'UTR; PRKCB, KLF10, KCND2, THRA, NFIA, COL11A1, FGF13, ATP1A2, MYH9, PCSK1, CUGBP2, EPHA7*

KROX family	NA	*BDNF pV, BDNF exon IV; PPP3CA, NFIA, DBN1, KCND2, IRS2, MAN1A2, CCND2, PVRL3, XIAP, DLGAP4, CYR61, ATP1B1, PURA, SMARCA4, MYH9, GRIA3, EPHA4, DUSP6, EGR1, COL4A5, TRPC4, PRKCB, NPTX1, PTGS2, EPHA5, FGFR1, CBX6, PRKCE, KLF10, THRA, ATP1A2, BAIAP2, CPD, CORO1A, CAMK2D, IGFBP5, DUSP1, PTPRF, FREQ, PRKAG2*

MAZ	NA	*BDNF pVh, BDNF exon III, IV; CD44, PPP3CA, PRKCE, COL4A5, USP2, PRKCB, KLF10, EPHA4, CAMK2D, CCND2, DUSP6, GRIA3, THRA, COL11A1, PENK, FGF13, CBX6, ATP1B1, PTPRF, ATP1A2, FREQ, DBN1, CUGBP2, EGR1, EPHA7*

MEF2	NA	*BDNF pII-V, BDNF exon II, IX, BDNF 3'UTR; CD44, TBX3, BAIAP2, PPP3CA, PRKCE, COL4A5, EPHA4, IL6ST, CAMK2D, CCND2, NEUROD6, DUSP6, MAN1A1, IGFBP5, COL11A1, TRPC4, PRSS23, ANGPT1, FREQ, PURA, MYH9, PCSK1, CUGBP2, EPHA7, SNCA, FGF13*

MYC/MAX	NA	*BDNF pI, II, IV; CD44, TBX3, PRKCE, USP2, CAMK2D, CCND2, NEUROD6, THRA, NFIA, DUSP1, CBX6, ATP1B1, FREQ, ITF-2, EGR1*

MYCN	NA	*BDNF pI, II; PRKCE, USP2, CAMK2D, CCND2, NEUROD6, THRA, DUSP1, CBX6, ATP1B1, FREQ, ITF-2*

MYOD	< 0.001	*BDNF exon I, IX; CD44, PRKCE, USP2, PRKCB, EPHA4, DUSP6, SCG2, SMARCA4, THRA, PRSS23, ATP1B1, CUGBP2*

NFkB	< 0.001	*BDNFI, BDNF 3'UTR; PPP3CA, KLF10, PCSK2, ATP1B1, ANGPT1, MYH9, USP2, DUSP6, FGF13, PURA, BAIAP2, CAMK2D, CCND2, FGFR1, CYR61, PCSK2, MYH9, CUGBP2, EGR1, EPHA7*

NRSF	NA	*BDNFII, EPHA4, IRS2, EPHA5, NPTX1, PRKCB, TRPC4, COL4A5*

S8	< 0.001	*BDNF pII-IV, BDNF exon II, IV, VIII, IX, BDNF 3'UTR; CD44, BAIAP2, PRKCE, NPTX1, EPHA4, CAMK2D, CCND2, NEUROD6, DUSP6, FGFR1, KCND2, MAN1A1, SCG2, THRA, NFIA, COL11A1, PENK, PCSK2, ANGPT1, PURA, ITF-2, MYH9, DBC1, CUGBP2, EGR1, EPHA5*

SOX5	0.001	*BDNF exon I, BDNF 3'UTR; EPHA4, THRA and PLAGL1 3'UTR; NFIA and OLFM1 promoters; SCG2 intergenic region; KCND2 intron*

TAL1/TCF4	NA	*BDNF pIV, BDNF exon I, BDNF 3'UTR; ATP1B1 3'UTR, MYH9 3'UTR and XIAP 3'UTR; SCG2, CD44, SERPINE1, SLC4A7, CCND2, NEUROD6, FGFR1, THRA, COL11A1, PCSK2, ANGPT1, DBC1, CUGBP2*

WT1	NA	*BDNF pI, BASP1, PPP3CA, NFIA, DBN1, EPHA7, BAIAP2, XIAP, DLGAP4, PURA, IRS2, ATP1B1, KCND2, GRIA3, HN1, EPHA4, EGR1, COL4A5, TRPC4, ATP1A2, PRKCB, NPTX1, DBC1, EPHA5*

In *BDNF*, TFBSs were found in promoters (p), exons or 3'UTR of the gene. In the correlated genes, TFBSs were searched for and discovered mostly in promoters (unless indicated otherwise). P-values are given for the TFBSs discovered using CONFAC. NA - not applicable for the TFBSs discovered using DiRE [see Additional files 10 and 11 for TFBS importance score].

To cross-check the results obtained with DiRE, we repeated the analysis using the CONFAC tool. CONFAC results overlapped with DiRE results and suggested novel regulatory elements in human *BDNF *promoters/exons I-IX and in *BDNF *3'UTR, which were highly conserved among mammals and over-represented in the BDNF-correlated genes. Then, evolutionary conservation across mammals was checked for the core element of each TFBS discovered in the *BDNF *gene using UCSC Genome Browser. Based on MW test results [see Additional file 12: The results of Mann-Whitney tests (CONFAC)], on the Importance score [see Additional file 10: DiRE motif discovery results for BDNF and 84 conserved correlated genes] and on the conservation data (UCSC), we propose potential regulators of *BDNF *(Figure 3 and Table 3) [see also Additional file 13: Highly conserved TFBSs in the *BDNF *gene (according to DiRE and CONFAC)]. It is remarkable, that the TFBSs discovered in the *BDNF *gene are highly conserved: most of the TFBSs are 100% conserved across mammals from human to armadillo, some of them being conserved even in fish (Figure 3).

## Discussion

Microarray meta-analysis has proved to be useful for constructing large gene-interaction networks and inferring evolutionarily conserved pathways. However, it is rarely used to explore the regulatory mechanisms of a single gene. We have exploited microarray data from 80 experiments for the purpose of the detailed analysis of the conservation of *BDNF *gene expression and regulation. Analysis of co-expression conservation combined with motif discovery allowed us to predict potential regulators of *BDNF *gene expression as well as to propose novel gene interactions. Several transcription factors that were identified here as potential regulators of human *BDNF *gene have been previously shown to regulate rodent *BDNF *transcription *in vitro *and *in vivo*. These transcription factors include REST (also known as NRSF) for *BDNF *promoter II [55], CREB for *BDNF *promoter I and IV [56,57], USF [58], NFkB [59], and MEF2 for *BDNF *promoter IV [60]. The support of the bioinformatics findings by experimental evidence strongly suggests that the potential regulatory elements discovered in this study in the *BDNF *locus may be involved in the regulation of *BDNF *expression.

According to g:Profiler, 44 out of 84 conserved correlated genes identified in this study (including *BDNF*) carry MYC-associated zinc finger protein (MAZ) transcription factor binding sites. Our study revealed putative binding sites for MAZ in *BDNF *promoter Vh and in exons III and IV, suggesting that MAZ could be involved in *BDNF *gene regulation from promoters III, and possibly from promoters IV, V, Vh and VI that lie in close proximity in the genome. It has been shown that MAZ is a transcriptional regulator of muscle-specific genes in skeletal and cardiac myocytes [61]. Histone deacetylation and DNA methylation might be involved in the regulation of expression of target genes by MAZ [62]. *BDNF *mRNA expression in the heart is driven by promoters IV, Vh and VI [11]. Epigenetic regulation of the *BDNF *gene expression is achieved in a cell-type and promoter-specific manner [12,63]. This could be a possible regulation mechanism of the *BDNF *gene by MAZ. Also, MAZ drives tumor-specific expression of *PPARG *in breast cancer cells, a nuclear receptor that plays a pivotal role in breast cancer [64]. Expression levels of *BDNF *and BDNF-correlated genes *CCND2, DUSP6, EGR1, KLF10 *and *PTPRF *are altered in breast cancer (see Table 2). These genes were identified as putative targets of MAZ in the present study suggesting potential role for MAZ in their regulation in breast cancer cells.

Our analysis revealed that Wilms' tumor suppressor 1 (WT1) transcription factor binding sites are overrepresented in the BDNF-correlated genes. WT1 binding sites were detected in *BDNF *promoter I, in *IRS2 *(insulin receptor substrate 2), *EGR1, BAIAP2 *(insulin receptor substrate p53) and *PURA *promoters and in 19 other genes. WT1 acts as an oncogene in Wilms' tumor (or nephroblastoma), gliomas [65] and various other human cancers [66]. WT1 activates the *PDGFA *gene in desmoplastic small round-cell tumor, which contributes to the fibrosis associated with this tumor [67]. Puralpha (*PURA*), a putative WT1 target gene identified in this study, has also been reported to enhance transcription of the *PDGFA *gene [68]. WT1 regulates the expression of several factors from the insulin-like growth factor signaling pathway [69]. WT1 was also shown to bind the promoter of *EGR1 *gene [70]. Neurotrophins and their receptors also may be involved in the pathogenesis of some Wilms' tumors [71]. Transcriptional activation of BDNF receptor *NTRK2 *by WT1 has been shown to be important for normal vascularization of the developing heart [72]. Moreover, WT1 might have a role in neurodegeneration, observed in Alzheimer's disease brain [73]. We hypothesize that *BDNF *and other WT1 targets identified in this study, can play a role in normal development and tumorigenesis associated with WT1.

KROX family transcription factors' binding sites were found to be abundant in the promoters of *BDNF *and BDNF-correlated genes. KROX binding motif was detected in *BDNF *promoter V and EGR2 binding site was found in *BDNF *promoter IV. Also, *EGR1 *gene expression was correlated with *BDNF *in human, mouse and rat. KROX family of zinc finger-containing transcriptional regulators, also known as Early Growth Response (EGR) gene family, consists of EGR1-EGR4 brain-specific transcription factors [74] that are able to bind to the same consensus DNA sequence (KROX motif) [75]. EGR1 is involved in the maintenance of long-term potentiation (LTP) and is required for the consolidation of long-term memory [76]. EGR3 is essential for short-term memory formation [77] and EGR2 is necessary for Schwann cell differentiation and myelination [78,79]. Since BDNF plays a significant role in the above mentioned processes, it would be intriguing to study the regulation of BDNF by EGR factors.

Binding sites for GFI1 and MEF2 were found in *BDNF *promoters, exons and 3'UTR, and in the promoter of the *SNCA *gene. GFI1 binding sites were detected in *BDNF *promoters II-VI and in exon I. MEF2 sites were found in *BDNF *promoters II-V and in exons II and IX. *SNCA *overexpression and gene mutations that lead to SNCA protein aggregation cause Parkinson's disease (PD) [80]. *BDNF *and *SNCA *expression levels change conversely in the nigro-striatal dopamine region of the PD brain [80,81]. The myocyte enhancer factor-2 (MEF2) is known to be necessary for neurogenesis and activity-dependent neuronal survival [82,83]. Inactivation of MEF2 is responsible for dopaminergic loss *in vivo *in an MPTP mouse model of PD [84]. MEF2 recruits transcriptional co-repressor Cabin1 and class II HDACs to specific DNA sites in a calcium-dependent manner [85]. MEF2 is one of the TFs that contribute to the activity-dependent BDNF transcription from promoter IV [60]. The growth factor independence-1 (GFI1) transcription factor is essential for the development of neuroendocrine cells, sensory neurons, and blood. Also, GFI1 acts as an oncogene in human small cell lung cancer (SCLC), the deadliest neuroendocrine tumor [86]. GFI1 mediates reversible transcriptional repression by recruiting the eight 21 corepressor (ETO), histone deacetylase (HDAC) enzymes and the G9a histone lysine methyltransferase [87]. It has also been shown that GFI1 Drosophila homolog Senseless interacts with proneural proteins and functions as a transcriptional co-activator suggesting that GFI1 also cooperates with bHLH proteins in several contexts [88]. Our findings are impelling to explore inverse regulation of *BDNF *and *SNCA *genes by GFI1 and MEF2 in neurons generally and in Parkinson's disease models in particular.

*BDNF *promoters II-V and *BDNF *exons II, IV and IX contain BRN2 (brain-specific homeobox/POU domain POU3F2) binding sequences. BRN2 is driving expression of the *EGR2 *gene - an important factor controlling myelination in Schwann cells [78,79]. BRN2 also activates the promoter of the Notch ligand Delta1, regulating neurogenesis. It also regulates the division of neural progenitors, as well as differentiation and migration of neurons [89]. Considering a prominent role of BDNF in myelination and neurogenesis, it is reasonable to hypothesize that BRN2 fulfills its tasks in part by regulating *BDNF *gene expression.

Evidence is emerging that not only proximal promoters, but also distant elements upstream and downstream from TSS can regulate transcription [90,91]. We found that *BDNF *3'UTR contains potential binding sites for TCF4 (also known as ITF2), GFI1, BRN2, NFkB and MEF2.

Finally, we have discovered multiple binding sites in human *BDNF *promoters for the transcription factors that have been shown to participate in neuronal activity-dependent transcription of rodent *BDNF *gene. *BDNF *promoters I and IV are the most highly induced following neuronal activation. *BDNF *promoter I was shown to be regulated by cAMP-responsive element (CRE) and the binding sequence for upstream stimulatory factor 1/2 (USF) in response to neuronal activity and elevated calcium levels [92]. Several TFs (USF [58], CREB [57], MEF2 [60], CaRF [93] and MeCP2 [63]) regulate *BDNF *promoter IV upon calcium influx into neurons. Rat *BDNF *promoter II has also shown induction by neuronal activity, though to a lesser extent compared to promoters I and IV [12,94]. However, calcium responsive elements have not been yet studied in *BDNF *promoter II and it was believed that its induction is regulated by the elements located in the promoter I. Our analysis of human *BDNF *gene detected CREBP1 and USF binding sites in *BDNF *promoter I, USF and MEF2 binding sites in promoter II and USF, MEF2 and CREB binding sites in promoter IV. We suggest that MEF2 and USF elements might contribute to *BDFN *promoter II induction by neuronal activity. In addition, we have detected conserved TCF4 (ITF2) binding sequences in *BDNF *promoter IV, and in exon I. It has been shown that calcium-sensor protein calmodulin can interact with the DNA binding basic helix-loop-helix (bHLH) domain of TCF4 inhibiting its transcriptional activity [95]. Preliminary experimental evidence (Sepp and Timmusk, unpublished data) suggests that TCF4 transcription factor is involved in the regulation of *BDNF *transcription. TCF4 might play in concert with CREB, MEF2 and other transcription factors to modulate *BDNF *levels following neuronal activity.

In our study we performed the analysis of a well-known gene and it served as a good reference to evaluate the results of the "subset" approach. However, the "subset" method coupled with the analysis of evolutionary conservation of co-expression is suitable for studying poorly annotated genes as well. This approach examines co-expression across a variety of conditions, which helps to discover novel biological processes and pathways that the guide-gene and its co-expressed genes are related to. Also, searching for conserved TFBS modules in co-expressed genes helps to discover functionally important genomic regions and this does not require detailed prior knowledge of the guide-gene's structure. However, when attempting to study less known genes, additional *in silico *analysis of genomic sequences using bioinformatics tools for prediction of promoters, TSSs and exon-intron junctions would be useful. Also, sequence alignment with co-expressed genes' promoters would be informative.

## Conclusion

A major impediment of meta-coexpression analysis is the differences among experiments. So far, analyzing gene expression across different microarray platforms remains a challenge. Discrepancies in the expression measurements among different platforms originate from different probe sequences used, different number of genes on the platform, etc. Therefore, in order to obtain reliable results, we used only one microarray platform type for the analysis. In addition, we introduced a new approach to increase the accuracy of the analysis: we divided datasets into subsets and sought for correlated genes for each subset, implying that each subset represents an independent experimental condition. We have also performed correlation link confirmation among subsets and correlation conservation analysis to discover functionally related genes.

One of the limitations of the co-expression conservation analysis is the fact that it detects only phylogenetically conserved co-expression events. Human-specific phenomena cannot be captured by this kind of analysis. In relation to *BDNF *this means, for example, that regulation of human *BDNF *gene by antisense *BDNF *RNA (*BDNFOS *gene) [11,96] could not be studied by co-expression conservation analysis, since BDNFOS gene is not expressed in rodents [12,97]. Also, co-expression analysis using microarray experiments is limited by the number of genes included in the microarray platforms. For example, since *BDNFOS *probe sets were absent from microarray platforms, we could not study co-expression, anti-coexpression or differential expression of *BDNF *and *BDNFOS*. In addition, our list of correlated genes did not include all possible correlation links with *BDNF *due to the fact that our analysis was deliberately limited to Affymetrix microarray platforms. Moreover, in our analysis we included only those experiments that met certain requirements regarding the *BDNF *gene expression. However, biologically meaningful results justify our rigorous filtering approach: correlated genes identified in this study are known to regulate nervous system development, and are associated with various types of cancer and neurological disorders. Also, experimental evidence supports the hypothesis, that transcription factor identified here can act as potential *BDNF *regulators.

In summary, we have discovered a set of genes whose co-expression with *BDNF *was conserved between human and rodents. Also, we detected new potential regulatory elements in BDNF-correlated genes and in the *BDNF *locus using bioinformatics analysis, in which *BDNF *was playing a role of a guide-gene. The presented concept of co-expression conservation analysis can be used to study the regulation of any other gene of interest. The study provides an example of using high-throughput advancements in studying single genes and proposes hypotheses that could be tested using molecular biology techniques.

## Methods

### Microarray datasets and data filtering

*Homo sapiens, Mus Musculus *and *Rattus Norvegicus *microarray datasets were downloaded from (GEO) [98]. We selected Affymetrix GeneChips experiments that comprised a minimum of 16 samples. Datasets which contained BDNF Detection call = Absent [99] in more than 30% of the samples were not selected [see Additional file 2: Microarray datasets] for the list of datasets used in the analysis. Since the arrays contained normalized data, no additional transformation was performed. To reduce the noise, we carried out non-specific filtering of data in each dataset. Genes that had missing values in more than 1/3 of the samples of a given dataset were excluded from the analysis in order to avoid data over-imputation [100]. For the remaining genes, we followed a column-average imputation method. Totally, only 0.098% of the gene expression values were imputed with this approach. Further, we selected the genes whose expression changes were greater than two-fold from the average (across all samples) in at least five samples in a dataset [19,49]. Additionally, datasets were eliminated from the study if *BDNF *probe sets' expression failed to meet the above mentioned criteria [see Additional file 1: BDNF probe sets]. Out of 72 human datasets, only 38 passed non-specific filtering, whereas 24 out of 82 mouse and 18 out of 35 rat datasets passed the filtering and were used for the analysis.

Each dataset was split into subsets (i.e. normal tissue, disease tissue, control, treatment, disease progression, age, etc.) so that subsets of the same dataset would not have any overlapping samples [see Additional file 3: Subsets]. The division into subsets was performed manually, according to the information included in the experiment. In some cases subsets could be further subdivided into biologically appropriate sub-subsets [see Additional file 2: Microarray datasets and Additional file 3: Subsets]. Subsets that contained less than eight samples were excluded from analysis to avoid inaccuracy in the estimation of genic correlations. Biological and technical replicates were handled as equal. From all human datasets, one (GDS564 dataset) contained one technical replicate per male sample and one technical replicate for all female samples except one. For the mouse datasets no technical replicates' data accompanied the dataset information. Finally, in rat GDS1629 dataset one technical replicate has been used for each biological replicate.

### Differential expression

We used Kruskal-Wallis test [23] to measure differential expression of BDNF across subsets in each dataset. Kruskal-Wallis test is a non-parametric method for testing equality of population medians within different groups. It is similar to one-way analysis of variance (ANOVA). However, it does not require the normality assumption. Alternatively, it represents an extension of Mann-Whitney U test [101,102] for more than 2 samples. Since we used multiple datasets we applied the false discovery rate approach (FDR) at the 0.05 level as it is described by Benjamini and Hochberg (1995) [103].

### Co-expression analysis

For each gene standard Pearson correlation coefficient (PCC) was calculated across samples. We followed a resampling strategy, which allows the calculation of the standard deviation of the PCC between a pair of probe sets. PCC was calculated for each subset separately. The PCC was calculated following a resampling bootstrap approach. For example, in order to calculate the CC_j _between *BDNF *and gene j when data consisted of m points, we resampled the m points with replacement creating 2000 re-samples [104]. Then the CC_j _was calculated as the average CC for the 2000 re-samples and the 95% bootstrap confidence interval was estimated. The average CC is very close to the sample CC. However, when m is a small number and outliers are contained in the sample then the bootstrap confidence interval may be large. The motivation behind the bootstrap approach is to avoid genes with large bootstrap confidence intervals. Thus, when we request the links between *BDNF *and the genes in the microarray experiment we ask for the genes j, whose CC_j _is greater than 0.6 and the 95% bootstrap confidence interval contains only positive numbers. If instead of the bootstrapping approach we would use just the sample CC, which is more efficient computationally, then a larger set of links would be obtained which would contain some genes with very large bootstrap confidence intervals.

A threshold value of r = 0.6 was used to retrieve a list of probe sets that were co-expressed with the *BDNF *probe set [22,49]. Each probe set correlation with *BDNF *that passed the threshold was termed as a "link". It should be noted that the PCC was calculated between probe set pairs and not between gene-name pairs. Thus, when more than one probe set-pair was associated with the same gene-pair we excluded all the links except the one with the highest PCC value.

### Co-expression link confirmation

We defined a "co-expression link confirmation" as a re-occurrence of links in multiple subsets. In order to avoid artifacts and biologically irrelevant links, we performed link confirmation to select the genes that were correlated with *BDNF *in three or more subsets [15]. It should be noticed that systematic differential expression within a subset could result in high PCC values. However, high PCC values in this case do not reveal any relationship between genes and represent a by-product of the differential expression of genes within a heterogeneous subset. We used a minimum between 1000 and 10% of all the probe sets within the subset as a threshold. Subsets that yielded more co-expression links between *BDNF *and other genes than an arbitrary threshold were excluded from further analysis. Thus, 5% of all the subsets were excluded.

### Probe set re-annotation and ortholog search

Prior to the identification of the links that are conserved between human, mouse and rat, we transformed the probe set-pair links to gene-pair links. We used g:Profiler [26] to transform the probe set names to Ensemble gene names (ENSG). However, since many probe sets are currently related to the expressed sequence tags (ESTs), not all the probe sets could be mapped to the known genes using g:Profiler. For each dataset, we used its annotation file (see: ). To assign Ensemble gene names to the "unmapped" probe sets, we obtained the probe set sequence identifier (GI number) using the annotation file. Then, we retrieved RefSeq accession for each GI number from NCBI database. Finally, we continued with a best-hit blast approach for all three species.

### Co-expression conservation and g:Profiler analysis

By performing a co-expression conservation analysis we identified the links that have passed prior filters (PCC threshold and link confirmation) and are conserved among human, mouse and rat.

Genes which co-expression with *BDNF *was found to be conserved between human, mouse, and rat constituted the input list for the g:Profiler. g:Profiler [26] is a public web server used for characterizing and manipulating gene lists resulting from mining high-throughput genomic data. It detects gene-ontology categories that are overrepresented by the input list of genes or by sorted sublists of the input. g:Profiler is using the "Set Count and Sizes" (SCS) method to calculate p-values [26].

### Correlated genes' interactions

We used iHOP resource (Information Hyperlinked over Proteins, ) [30] to find reports in the literature about known interaction between BDNF-correlated genes. iHOP generates a network of genes and proteins by mining the abstracts from PubMed. A link in such a network does not mean a specific regulatory relationship, but any possible interaction between two genes (such as protein activation, regulation of transcription, co-expression, etc). Each reference was verified manually to ensure the citation of valid interactions.

### Motif discovery

We clustered BDNF-correlated genes according to their tissue-specific expression using gene expression information available in the TiProD database [51] (*BDNF *gene was included in every cluster). The TiProD database contains information about promoter tissue-specific expression for human genes. For each gene the list of tissues where the gene expression has been detected can be obtained from TiProD together with the tissue specificity score. For each gene we extracted information on tissue expression, selecting tissues with specificity score higher than 0.2. Each tissue was assigned a category according to its anatomy and function and the genes expressed in corresponding tissues were clustered into CNS, peripheral NS, endocrine, gastrointestinal or genitourinary cluster. Then, we searched for combinations of over-represented TFBS among the list of correlated genes, as well as the tissue clusters discovered by TiProD.

We used DiRE [52] and CONFAC [53] tools for the discovery of TFBSs in the conserved co-expressed genes. DiRE uses position weight matrices (PWM) available from version 10.2 of the TRANSFAC Professional database [105]. In DiRE, up to 5000 background genes can be used. Only those TFBSs are extracted that occur less frequently in 95% of permutation tests than in the original distribution (corresponding to a *p*-value < 0.05 to observe the original distribution by chance) and that corresponds to at least a twofold increase in their density in the original distribution as compared with an average pair density in permutation tests. To correct for multiple hypothesis testing, the hypergeometric distribution with Bonferroni correction is used in the DiRE tool [106]. For each discovered TFBS DiRE defines the 'importance score' as the product of the transcription factor (TF) occurrence (percentage of tissue-specific TF with the particular TFBS) and its weight (tissue-specificity importance) in a tissue-specific set of candidate TF. Thus, the importance score is based on the abundance of the TFBS in tissue-specific TF and on the specificity of the TF that contain the particular TFBS.

Conserved transcription factor binding site (CONFAC) software [53] enables the high-throughput identification of conserved enriched TFBSs in the regulatory regions of sets of genes using TRANSFAC matrices. CONFAC uses the Mann-Whitney U-test to compare the query and the background set. It uses a heuristic method for reducing the number of false positives while retaining likely important TFBSs by applying the mean-difference cutoff which is similar to the use of fold change cutoffs in SAM analyses [107] of DNA microarray data [53]. According to the data provided by CONFAC, 50 random gene sets were compared to random sets of 250 control genes. Only one TFBS exceeded 5% false positive rate for the set of 250 random control genes that we used in our analysis with the parameters advised by the authors [53]. We used promoter sequences of BDNF-correlated genes and the sequences of *BDNF *promoters, exons, introns and the 3'UTR for the analysis. Matrix Similarity cut-off 0.85 and Core Similarity cut-off 0.95 were used for motif discovery; and the parameters recommended by authors - for Mann-Whitney tests (*p*-value cutoff 0.05 and mean-difference cutoff 0.5) [53].

Evolutionary conservation across mammals was confirmed manually for the 5-nucleotide core element of each TFBS discovered in the *BDNF *gene using UCSC Genome Browser [108].

## Authors' contributions

TA and PP made equal contribution to conception and design of the study. PP performed computational analysis of data; TA and TT performed interpretation of the results. TA and PP were involved in drafting the manuscript; TT revised the manuscript for important intellectual content. TA, PP and TT have given final approval of the version to be published.

## Supplementary Material

Additional file 1 **BDNF probe sets**. Affymetrix microarray probe sets for BDNF gene. BDNF probe set target sequences are given for each platform type that was used in the co-expression conservation analysis. Click here for file

Additional file 2 **Microarray datasets**. Datasets that passed non-specific filtering and were used in the analysis (38 human microarray datasets, 24 mouse datasets and 18 rat datasets). Each dataset was divided into subsets (disease state, age, agent, etc) according to experimental annotations. When possible, subsets were subdivided further (marked by *). Experiments were classified based on their description and the tissue origin. GDS refers to GEO Datasets. Click here for file

Additional file 3 **Subsets**. Dataset: GDS1018/1368678_at/Bdnf/Rattus norvegicus. Expression profiling of brain hippocampal CA1 and CA3 neurons of Sprague Dawleys subjected to brief preconditioning seizures. According to the dataset annotation, dataset could be divided into three subsets by cell type (A) or into two subsets by protocol (B). In addition, subsets could be subdivided further into cell type.protocol sub-subsets: CA1 pyramidal neuron.control, CA1 pyramidal neuron.preconditioning seizure, CA3 pyramidal neuron.control, etc. After filtering, subset containing less than eight samples (CA3 pyramidal neuron.preconditioning seizure) was excluded from the analysis. Click here for file

Additional file 4 **Differential expression of the BDNF gene in human datasets**. Differential expression of BDNF was measured across subsets in each dataset using Kruskal-Wallis test. Only statistically significant results are presented. Click here for file

Additional file 5 **Differential expression of the BDNF gene in mouse datasets**. Differential expression of BDNF was measured across subsets in each dataset using Kruskal-Wallis test. Only statistically significant results are presented. Click here for file

Additional file 6 **Differential expression of the BDNF gene in rat datasets**. Differential expression of BDNF was measured across subsets in each dataset using Kruskal-Wallis test. Only statistically significant results are presented. Click here for file

Additional file 7 **Conserved BDNF-correlated genes**. Genes, whose correlation with BDNF was confirmed in at least 3 subsets (3+ genes) and was conserved between i) human, mouse and rat; ii) human and rat; iii) human and mouse; iv) mouse and rat. Click here for file

Additional file 8 **g:Profiler analysis**. Functional profiling of the list of BDNF-correlated genes conserved between human, mouse and rat using g:G:Profiler. For details see also . Click here for file

Additional file 9 **iHOP references**. Interactions between conserved correlated genes in human and mouse (URL links to the literature cited in iHOP). Click here for file

Additional file 10 **DiRE motif discovery results for BDNF and 84 conserved correlated genes**. Over-represented TFBSs are given together with the Importance Score (cut-off 0.1 recommended by DiRE). Numbers 1 and 2 (in All 1 and 2) refer to the different ways that DiRE tool analyzes evolutionary conserved regions (ECR): 1) top 3 ECRs + promoter ECRs; 2) UTR ECRs + promoter ECRs. Click here for file

Additional file 11 **DiRE motif discovery results for conserved BDNF-correlated genes clustered by tissue-specific expression**. TFBSs over-represented in each tissue cluster are given together with the Importance Score (cut-off 0.1 recommended by DiRE). CNS - central nervous system, PNS - peripheral nervous system. Click here for file

Additional file 12 **The results of Mann-Whitney tests (CONFAC)**. Overrepresented TFs in the conserved BDNF-correlated gene list. Bar graphs show the average conserved TFBS frequencies for the sample gene set (conserved BDNF-correlated genes, blue bars) and control gene set (random 250 genes, red bars). A minimum threshold for the differences in the average TFBS frequencies between the two groups was set by p-value cutoff 0.05 and a mean-difference cutoff 0.5. Click here for file

Additional file 13 **Highly conserved TFBSs in the BDNF gene (according to DiRE and CONFAC)**. Represented TFBSs have Matrix Similarity score >0.85 and Core Similarity score >0.99. TFBS sequences are highlighted in blue; "+" or "-" mark the DNA strand orientation; BDNF exons and 3'UTR are highlighted in green; the regulatory region in BDNF downstream from polyadenylation sites identified by DiRE is highlighted yellow. Click here for file
