# Supplementary material for: Meta-coexpression conservation analysis of microarray data: a "subset" approach provides insight into brain-derived neurotrophic factor regulation
Source: BMC Genomics. 2009 Sep 8;10:420. doi: 10.1186/1471-2164-10-420 (PMC2748098; doi:10.1186/1471-2164-10-420)
Supplement: Additional file 3 — Subsets. Dataset: GDS1018/1368678_at/Bdnf/Rattus norvegicus. Expression profiling of brain hippocampal CA1 and CA3 neurons of Sprague Dawleys subjected to brief preconditioning seizures. According to the dataset annotation, dataset could be divided into three subsets by cell type (A) or into two subsets by protocol (B). In addition, subsets could be subdivided further into cell type.protocol sub-subsets: CA1 pyramidal neuron.control, CA1 pyramidal neuron.preconditioning seizure, CA3 pyramidal neuron.control, etc. After filtering, subset containing less than eight samples (CA3 pyramidal neuron.preconditioning seizure) was excluded from the analysis. [file 1471-2164-10-420-S3.pdf]

A

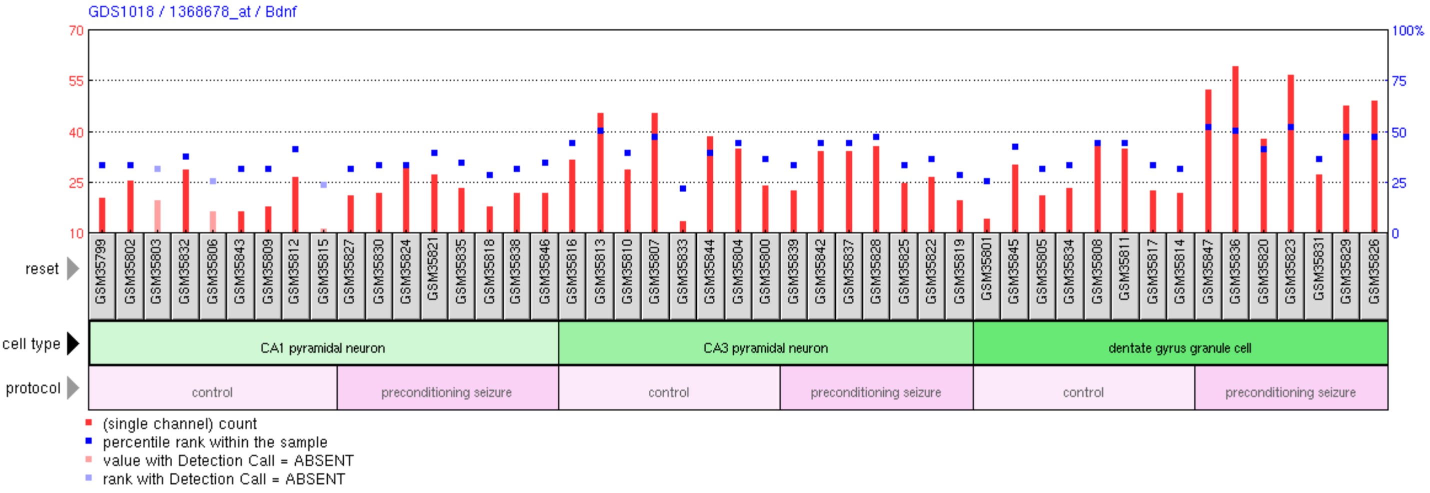

B

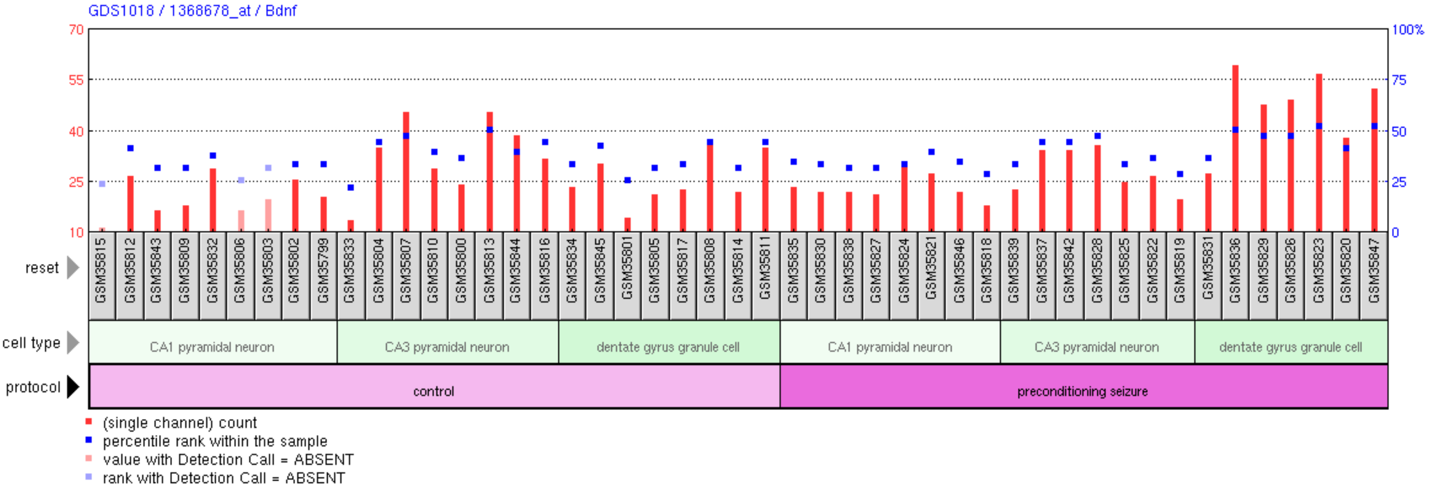

Additional file 3. Subsets
Dataset: GDS1018 / 1368678_at / Bdnf / *Rattus norvegicus*. Expression profiling of brain hippocampal CA1 and CA3 neurons of Sprague Dawleys subjected to brief preconditioning seizures. According to the dataset annotation, dataset could be divided into three subsets by cell type (A) or into two subsets by protocol (B). In addition, subsets could be subdivided further into cell type.protocol sub-subsets: CA1 pyramidal neuron.control, CA1 pyramidal neuron.preconditioning seizure, CA3 pyramidal neuron.control, etc. After filtering, subset containing less than eight samples (CA3 pyramidal neuron.preconditioning seizure) was excluded from the analysis.
