# Supplementary material for: Meta-coexpression conservation analysis of microarray data: a "subset" approach provides insight into brain-derived neurotrophic factor regulation
Source: BMC Genomics. 2009 Sep 8;10:420. doi: 10.1186/1471-2164-10-420 (PMC2748098; doi:10.1186/1471-2164-10-420)
Supplement: Additional file 4 — Differential expression of the BDNF gene in human datasets. Differential expression of BDNF was measured across subsets in each dataset using Kruskal-Wallis test. Only statistically significant results are presented. [file 1471-2164-10-420-S4.pdf]

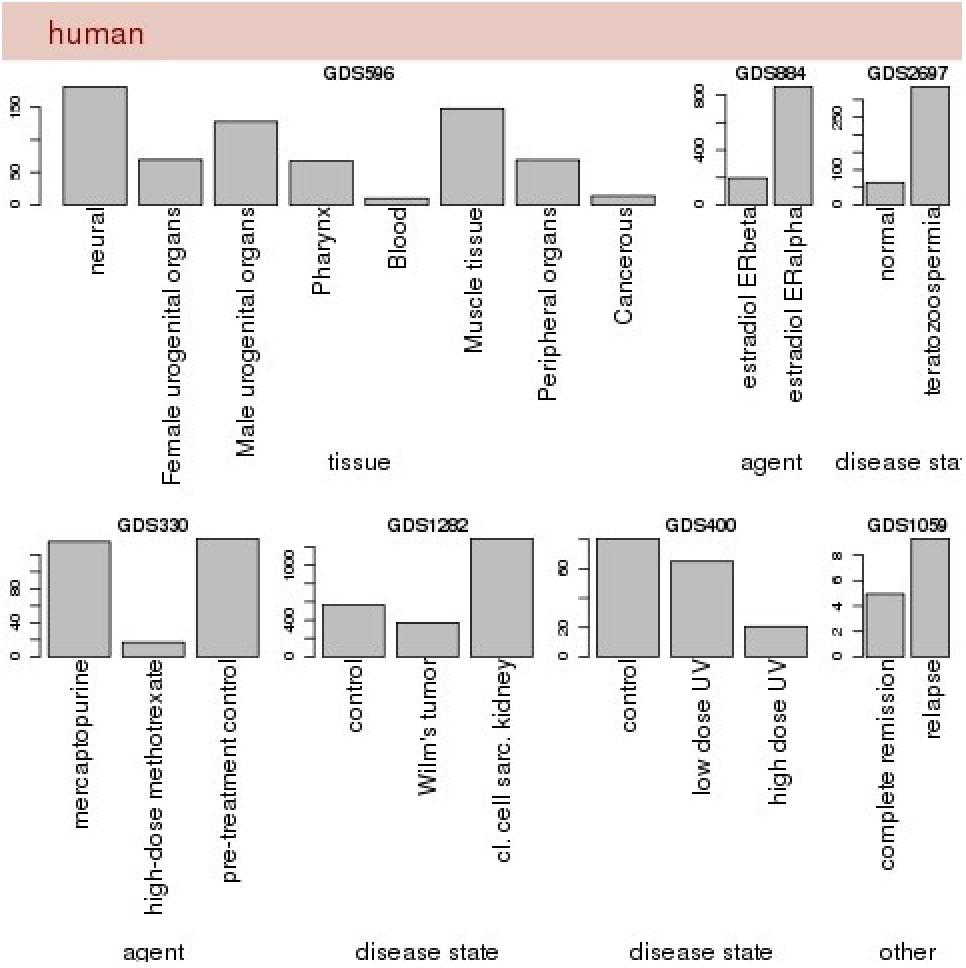


Additional file 4. Differential expression of the BDNF gene in human datasets.
Differential expression of BDNF was measured across subsets in each dataset using Kruskal-Wallis test. Only statistically significant results are presented (false discovery rate 0.05). Each bar represents median expression value of BDNF in a given subset. GDS stands for GEO dataset accession number. GDS1282: cl. cell sarc. kidney - clear cell sarcoma of the kidney.
