# Supplementary material for: Meta-coexpression conservation analysis of microarray data: a "subset" approach provides insight into brain-derived neurotrophic factor regulation
Source: BMC Genomics. 2009 Sep 8;10:420. doi: 10.1186/1471-2164-10-420 (PMC2748098; doi:10.1186/1471-2164-10-420)
Supplement: Additional file 5 — Differential expression of the BDNF gene in mouse datasets. Differential expression of BDNF was measured across subsets in each dataset using Kruskal-Wallis test. Only statistically significant results are presented. [file 1471-2164-10-420-S5.pdf]

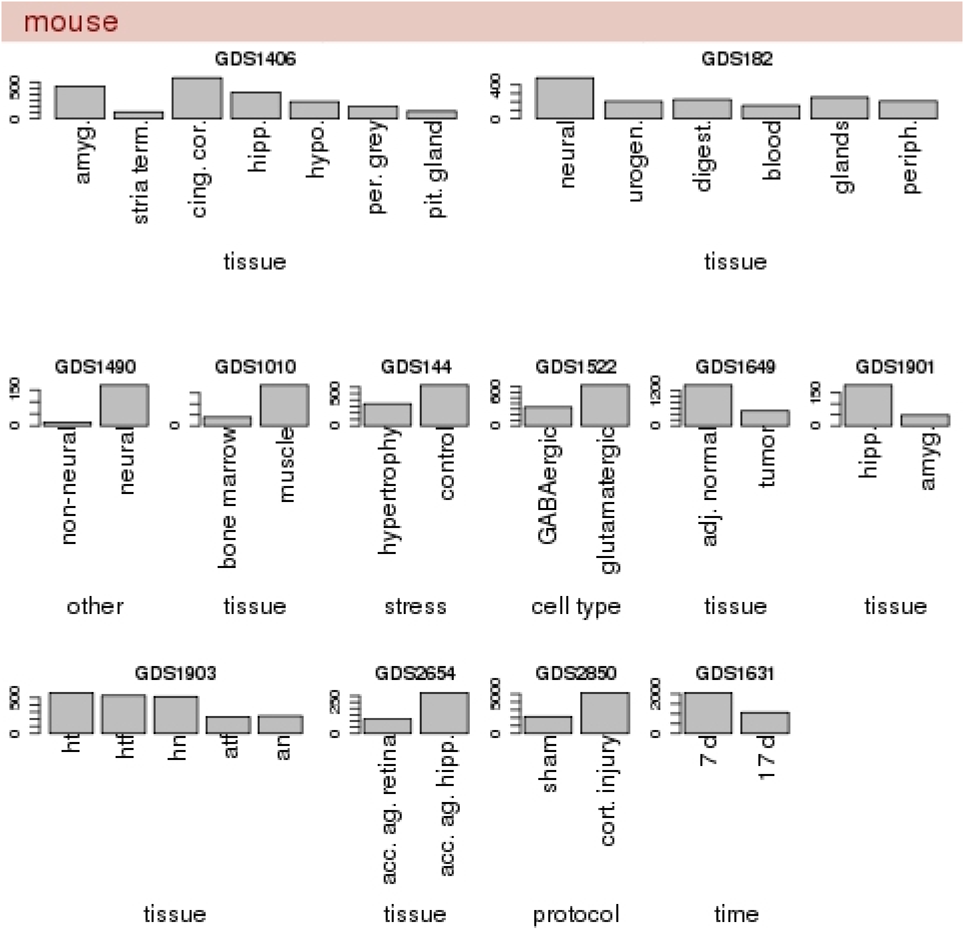

Additional file 5. Differential expression of the BDNF gene in mouse datasets.
Differential expression of BDNF was measured across subsets in each dataset using Kruskal-Wallis test. Only statistically significant results are presented (false discovery rate 0.05). Each bar represents median expression value of BDNF in a given subset. GDS stands for GEO dataset accession number. GDS1406: amyg - amygdala; stria term. - bed nucleus of stria terminalis; cing. cor. - cingulate cortex; hipp. - hippocampus; hypo. - hypothalamus; per. grey - periaqueductal grey; pit. gland - pituitary gland. GDS182: urogen. - urogenital system; digest. - digestive system; periph. - peripheral organs. GDS1649: adj. normal - adjacent normal. GDS1901: hipp. - hippocampus; amyg. - amygdala. GDS1903: ht - hippocampus, conditioning chamber, tone; htf - hippocampus, conditioning chamber, tone, footshock; hn - hippocampus, naive; aft - amygdala, conditioning chamber, tone, footshock; an - amygdala naive. GDS2654: acc. ag. retina - accelerated aging retina; acc. ag. hipp. - accelerated aging hippocampus. GDS2850: cort. injury - lateral control cortical impact injury.
