# Supplementary material for: Meta-coexpression conservation analysis of microarray data: a "subset" approach provides insight into brain-derived neurotrophic factor regulation
Source: BMC Genomics. 2009 Sep 8;10:420. doi: 10.1186/1471-2164-10-420 (PMC2748098; doi:10.1186/1471-2164-10-420)
Supplement: Additional file 6 — Differential expression of the BDNF gene in rat datasets. Differential expression of BDNF was measured across subsets in each dataset using Kruskal-Wallis test. Only statistically significant results are presented. [file 1471-2164-10-420-S6.pdf]

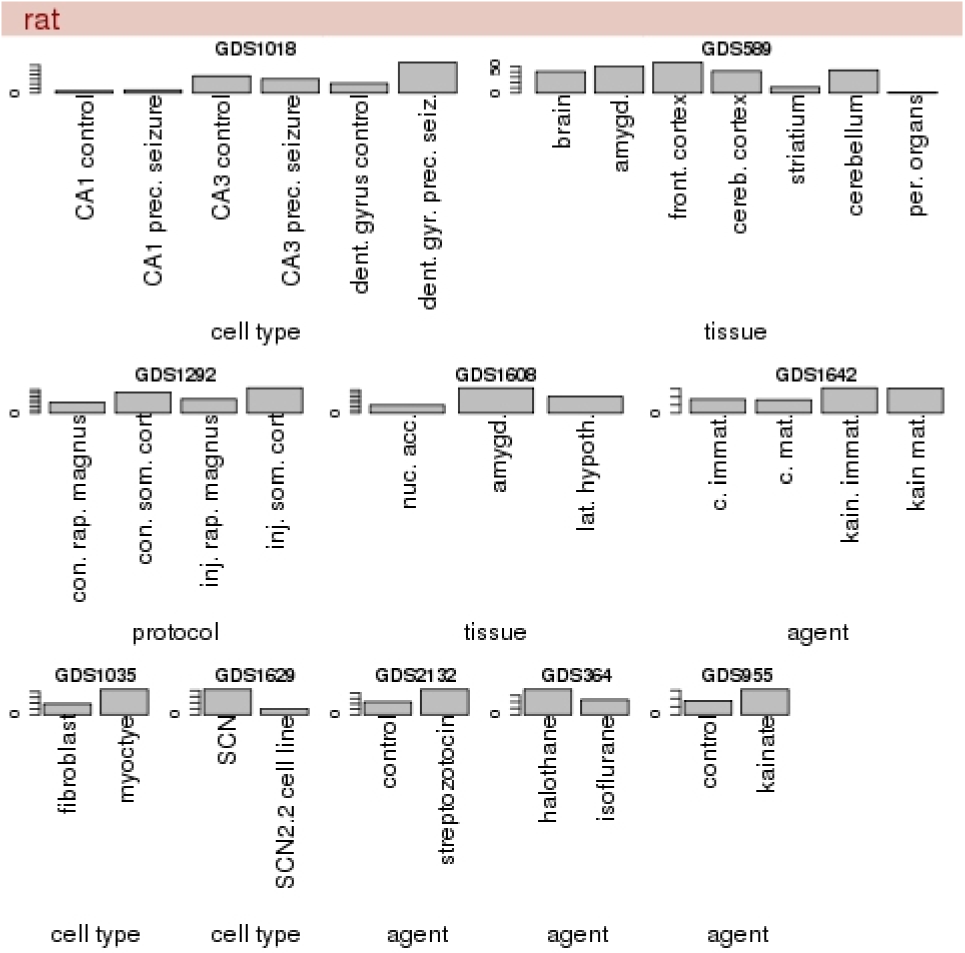

Additional file 6. Differential expression of the BDNF gene in rat datasets.
Differential expression of BDNF was measured across subsets in each dataset using Kruskal-Wallis test. Only statistically significant results are presented (false discovery rate 0.05). Each bar represents median expression value of BDNF in a given subset. GDS stands for GEO dataset accession number. GDS1018: prec. seizure - preconditioning seizure. GDS589: amygd - amygdala; front. cortex - frontal cortex; cereb. cortex - cerebral cortex; per. organs - peripheral organs. GDS1292: con. rap. magnus - control raphe magnus; con. som. cort. - control somatomotor cortex; inj. rap. magnus - moderate spinal cord injury raphe magnus; inj. som. cort. - moderate spinal cord injury somatomotor coretx. GDS1608: nuc. acc. - nucleus accumbens; amygd. - amygdala; lat. hypoth. - lateral hypothalamus. GDS1642: c. immat. - control immature; c. mat. - control mature; kain. immat. - kainate immature; kain. mat. - kainate mature.
