# Supplementary material for: Meta-coexpression conservation analysis of microarray data: a "subset" approach provides insight into brain-derived neurotrophic factor regulation
Source: BMC Genomics. 2009 Sep 8;10:420. doi: 10.1186/1471-2164-10-420 (PMC2748098; doi:10.1186/1471-2164-10-420)
Supplement: Additional file 12 — The results of Mann-Whitney tests (CONFAC). Overrepresented TFs in the conserved BDNF-correlated gene list. Bar graphs show the average conserved TFBS frequencies for the sample gene set (conserved BDNF-correlated genes, blue bars) and control gene set (random 250 genes, red bars). A minimum threshold for the differences in the average TFBS frequencies between the two groups was set by p-value cutoff 0.05 and a mean-difference cutoff 0.5. [file 1471-2164-10-420-S12.pdf]

Additional file 12. The results of Mann-Whitney test (CONFAC)

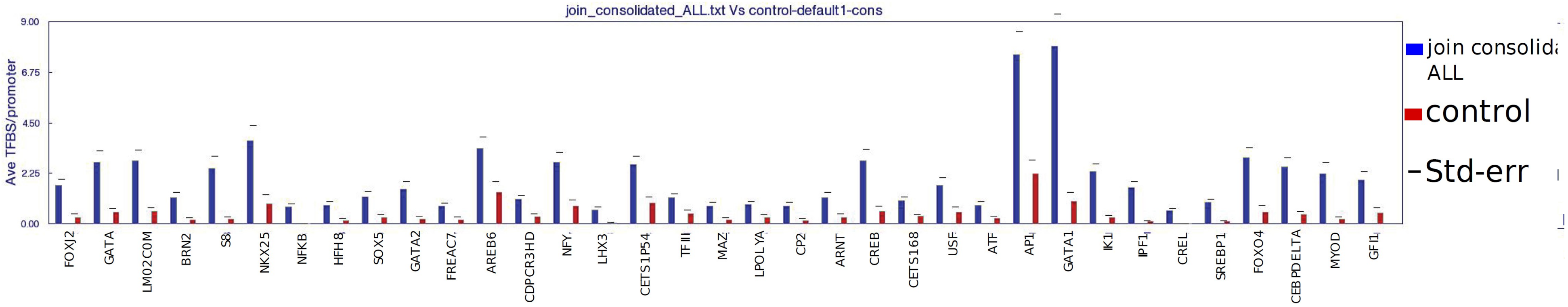

Overrepresented TFs in the conserved BDNF - correlated gene list. Bar graphs show the average conserved TFBS frequencies for the sample gene set (conserved BDNF-correlated genes, blue bars) and control gene set (random 250 genes, red bars). A minimum threshold for the differences in the average TFBS frequencies between the two groups was set by p-value cutoff 0.05 and a mean-difference cutoff 0.5.
