# Supplementary material for: Meta-coexpression conservation analysis of microarray data: a "subset" approach provides insight into brain-derived neurotrophic factor regulation
Source: BMC Genomics. 2009 Sep 8;10:420. doi: 10.1186/1471-2164-10-420 (PMC2748098; doi:10.1186/1471-2164-10-420)
Supplement: Additional file 13 — Highly conserved TFBSs in the BDNF gene (according to DiRE and CONFAC). Represented TFBSs have Matrix Similarity score >0.85 and Core Similarity score >0.99. TFBS sequences are highlighted in blue; "+" or "-" mark the DNA strand orientation; BDNF exons and 3'UTR are highlighted in green; the regulatory region in BDNF downstream from polyadenylation sites identified by DiRE is highlighted yellow. [file 1471-2164-10-420-S13.htm]

S8


**Additional file 13.  Highly conserved TFBSs in the
BDNF gene (according to DiRE and CONFAC results)**

Represented TFBSs have Matrix Similarity score >0.85 and
Core Similarity score >0.99.  TFBS sequences are highlighted in turquoise;
�+� or �-� mark the DNA strand orientation; BDNF exons and 3�UTR are
highlighted in green; the regulatory region in BDNF downstream from
polyadenylation sites identified by DiRE is highlighted yellow.

 

 

BDNF
cluster I

                                                              
IK1-

                                                            
NFkB+/-

CAGAGCGAGTAAAATCATGGTGTCAGTGCCAAAACGACGAAACCTGAAATGCGTCTGGGGACTTCCCAGGGCACTAGCGCTCCTCAGAGCGACGGACATC          
100

TCCATAACAACTGGTCTACATTCATCCCTGGTTCTTCTGCTCTGCTGTGCTAGCGATGGCTGAGGGTAGGCAAAGGGCAGCTTCAGTGGAAAGATACCTT          
200

GCGTTAGCGCCTCCCTAGGCTGGGGGACGTTGCCAAAGCTCGAGTTATTGCCTATTTCCCGAGATTTCCGGAGTCGTCCCCACATCTCCCGCGGAGAGCG          
300

GCGCCCCTCCGCCCCCCCTCCCGCCTCCCCCTCTATTAGACACTCCAAGGGGGTAGTTTGTTTCATTGTGCTTTTTTTCCTCTTCTCAAAGTTCATCCTT          
400

              
       MYCMAX+/-

                      
ARNT+/-

                        
NMYC+/-

                         
USF+/-                                                              
WT1+

TCGCCTCCAAGTTACTTTCCGCCAACACGTGACCTCTTCGCTTCCCAGCTTGCGCAGCCACTGGTGGGGAAGCGCGGGCGCTAACCGCAGGGCTTCCTGG          
500

GAGAGCCCCCTCCGAGGTTCGCCACCCCAGCCCTGGCTCCCGCCGCGCTCACCCCGGCACCCCGGCGGCGGGGCAGCCCCCGCAGGATGAGGAAGCGGCT          
600

CCGGGGAAGCAGCACCGAGCAGCAGCGGAGCGCCCAACCTGCCGCCTCCCCTCCCGTCCCTGCCTTGGCCCGCGGCTCCGCTCTCCGCGGCTCGCCTTCC          
700

GACCTAGCTGCGCACCGGGGCTGTTAACTCACATTTGGGAAGCCATAACCCATTAGAGCAAACGCAGTCATAACTTCATTCAACTCAGCCGCTCGAGAGC          
800

                                                                              
IK1+    

TCGGCTTACACAGGTTCCTGTGGGCAACTAGTGGCTCGCCTTGGTGCCTCTCGCCTAGTCATCAGTACCTAAGAGGAAAAGGGAAAGTTGTTGGGCTGGT          
900

                       
IK1-                

TCGCGCTTCGACGCATGCAGATGTTCCCAAGGACAAGTCACTTACTCGCCCCCCTCCCCCCAGTCCCCATTTGATCATCACTCACGACCTCATCGGCTGG          
1000                                                        

                
                                                                        CREBP1-

AGACCCTTAGTCATGATGGGGGAGGGGGAGGGGCACGAACTTTTCTAAGAAGTTTCCTTTTTTTACCCAGAGAGTCACAGTGAGTCGGTCACGTAAACAG          
1100

                        
ZNF219+                        
MRF2-     
                   GATA1+

CGAGGTTAGTCGTCGCCGTTGCCGCCCCCCACCCCCTCCCTGCTGCGCTTTTCTGGTATTATTATTAAAGCGGTAGTCTGCCGGCGCTGATAAGCAACAA          
11200                         
                    

 IK1-         
IK1-                                                        
                        

GTTCCCCAGCGGTCTTCCCGCCCTAGCCTGACAAGGCGAAGGTTTTCTTACCTGGCGACAGGGAAATCTCCCGAGCCGAATTCAGCTTCGCCGGAGCCCC          
1300

MYOD+                                                                                   
IK1+      

AGGTGTGACCTGCGTAGTGGGCAAGGGAGCGGTGTGCAGGCTGAGTTTTTTTTTTTACAGGGGTACCCTGAAACTCCTCACTTTCTCTGGGAACTTTCAG          
1400

                                                                                            
GFI1+ 

TGCCAGGACCCAGTAACGGGCGGTTAGAAGGCAGCCCTAGGAAACACCTGCTACATAGCAGGGCAGTTGGGCAATCATTGGTAACCTCGCTCATTCATTA          
exon I

   
CREBP1-                                                                                        

GAATCACGTAAGAACTCAAAAGGAAACGTGTCTCTCGGAGTGAGGGCGTTTGCGTAAATCTATAGGTTTTTCGACATCGATGCCAGTTGCTTTGTCTTCT          
1600

         
                           CHOP-                                                         

                               
GATA1+                                                             

GTAGTCGCCAAGGTGGTTGAGAGTTTAAGCTTGCGGATATTGCAAAGGGTTATTAGATTCATAAGTCACACCAAGTGGTGGGCGATCCACTGAGCAAAGC          
1700

                       
FOXO4+            
TAL1beta/ITF2-                                          
 

CGAACTTCTCACATGATGACTTCAAACAAGACACATTACCTTCCAGCATCTGTTGGGGAGACGAGATTTTAAGACACTTGAGTCTCCAGGACAGCAAAGG          
1800    
                                          

                                                                     
GATA1-                       

SOX5+                            S8-         
OCT1+               
OCT1-                    
SREBP1+

CACAATGGTGAGTAGCAATAAAACCTGCATTATAATTGAAAAATCTTGACATGTTGCTTAACAACGGGCATATCACGGCTCTTCCTAGCACTTCACACGC          
1900

                                                        
S8-    GFI1+     

CAAAGAACAGCAGCTACTCAGGCCAGGGGAATCGGGTTTTTACACAGTGCAACTTTAATTGGAATCATTTGAGATTTGACACAGCTATGTGGAACTGCGT          
2000

GGAACAAACTTGGAGCTGGGTGGGGGGGTGTGTGTTATATTGGTTGTTCAAGGCTGATGCTTGTCTCTCAGCAGTCTTGCATTCTATTCTTTTCCTTAAT          
2100

               
OCT1+                          
OCT1+                  

                      
GFI1-                 
S8-   
                         MMEF2+

GTGTATGGTGTATGATCATATTCTATGATTTATATGTGGGCATGTAATTGACATTTGCAAGGGGGTTAATTTCCATCTAAAAACAATAATGCTGTTAGAG          
2200

GTTGGGGTTAGGGGGTGGAGTGGGGGTAAGGGTGGGGTAAAGACTGGGAGTTTAGGTGTAGATGGGGGGTGGGGTTGGGGGGAGAGAAATAAGTCAGAAG          
2300

         
                                                                              SREBP1+/-

                                                                                        
MYCMAX+/-

                                                                    
                     ARNT+/-

                                                                                           
NMYC+/-

                  
BRN2+                                                                     
USF+/-

TGCATATCACCGGTAATGGGTAATCCTCTCGTAGAAGAAAAGGTTCTCATCAACATGTGATCAACTATTAACAGGATGGCTTTGGCAAAGCCATCCGCAC          
2400

                                                        
BRN2-

            
ETS2+                     
GATA1-            
S8+/-      

GTGACAAACCGTAAGGAAGTGGAAGAAACCGTCTAGAGCAATATCAAGTATCACTTAATTAGAGATTTTTAAGCCTTTTCCTCCTGCTGTGCCGGGTGTG          
2500

                             
NRSF+                                                                

TAATCCGGGCGATAGGAGTCCATTCAGCACCTTGGACAGAGCCAACGGATTTGTCCGAGGTGGCGGTACCCCCAGGTAGTCTTCTTGGCCCCGCTGTAAA          
exon II

                   
MMEF2+                                                                         

GCCAACCCTGTGTCGCCCTTAAAAAGCGTCTTTTCTGAGGTTCGGCTCACACTGAGATCGGGGCTGGAGAGAGAGTCAGATTTTGGAGCGGAGCGTTTGG          
2700

AAAGCGAGCCCCAGTTTGGTCCCCTCATTGAGCTCGCTGAAGTTGGCTTCCTAGCGGTGTAGGCTGGAATAGACTCTTGGCAAGCTCCGGGTTGGTATAC          
2800

             
OCT1+     
XFD2-                                          

        
IK1+       FOXO4-  
GATA1-                                     

TGGGTTAACTTTGGGAAATGCAAGTGTTTATCTCCAGGATCTAGCCACCGGGGTGGTGTAAGCCGCAAAGAAGGTAAGCACCAGGGCGGGGACCCCTTGC          
2900

                   
GATA1+

                
MEF2+

ATCCCCAATTCTTGAGCTATTTTGATACTGTCTTCCGGAGAGGACGCGTGGTGGAGGGGAGGAGGTAGAGGGAGAGCATGAGAGGGGGTTGTTTCTTGGT          
3000

               
                                                                GATA1+

ATTTGCCCAGTTTGAATTGCCCTAGGTGAGAACCCTGGGGCAAAGGGAGAAAGAAAAAAAAGAAACTCAGTCTTCCTGCGGATATAATGAGTTTAGTTAA          
3100

    
OCT1+     
GFI1-         GFI1-   

CTTGGACCTGCAAATGTCTGATTCAAATGTAAGATTTATCTCTCTTTTTCTCCTCTTCACCTCCCTCTTTTCCGTTCTCTTTGCTGGTGTGTGTGTGTGT          
3200

            
BRN2+/-

                    
S8+/-            
FOXO4+

GTGTACAGTAGATTCATTACTAATTATGAAGCTTTTGCAAAACATTCGAATTCCTAAAATTTGACTTTGTAGCATTTAGAATCAGGCGGTGGAGGTGGTG          
3300

            
                                                           GFI1+        
S8-

TGCGGTGGGGAGAGGAGGTGGAGGTTGGGAAGAGGGAAGGAGGTAAAGCTAAACCTCCAACACAAAAAAATGAATCAAGGTAATTTCAGCTCTTCTAGTG          
3400

AGAAGGATTCATTCTCTCTGTATCCCTCCCTCCCTCTCTTTCCCCCTCCCTCCCTCCTTCCCGCCCCCCTTCTTCCACCCCGCCCCCTCCTCCAGCCTCC          
3500

ATCCCTCCCTCATTCTATCTCTTCCTCTCCGTCGCCCTCGCTCCTCGCTGGATGCTTCTTTCTGGGTTTTCTTTTTTTTTTCCCTTCTGTCCTCCCTCCC    
     exon III

                                           
IK1+                        
MAZ-                      

CGCGAGTTTCGGGCGCTGGCTTAGAGGGTTCCCGCTTTCTCAAGGGAAGGGGAGCTGCCGAGACCGCGCTCCGCTCCCCAGCCGGGCCGGATGCCTCACT          
3700

GAGCCCAG

 

 

 

 

 

 

 

 

BDNF
cluster II

 

CCAACAATTTGCAAAGGCGCTCTGAATGATCACACATTCTGATAACACTTCCAAGGAACAGATAGCTTCACTTAGGGGGTGGGGGAGATGGAAGCAGGGT          
100

TATTTCTAGCAGGAATTCTTGAGTTCACTGAAGTCTTGTCCCTGGTACTTCACTGTGTGAACGTGGGTAAATTATTTCCTGGCAGAGGATCGGATTCTTC          
200

                                                
MEF2+    

TTTTATAAAACGGGTAAATAATTTCTGTCACTAGTCTTTAGAAGTTCTAAAATAGCTAATGTTAGTGAATTCATTTTGCTAACTGTAAACCCTTAGGTAA          
300

                           
OCT1+

ATTGAACTGAGTATGTAATAATATTATATATTCAGTTCAACAGCACATTCTTGGTAACCACAAGAGGGTCCAGGAAAGGAAACTGTTTATAAATCTTTCC          
400

CTTTAGCAAAATTAATGTTGGAGTCTTTAGGGAAATTCTTACAGCAATAGTCTTCGCAATTATTAGGTCAAACCCCTTTGAGATTACAGAAAAACGCACA          
500

CACACAGAAAGCTGCCTGCAGAATTTGGGTGTGGGCTTGGTGGGAGATTCCTCTGATACCCAGTGTTGTACCCCCAAGAGAGTGTTTCTCAAAGTGTGAC          
600

TTCAGATTGTCTGCATTCGAATTGCTTGTGGTATTTATTAAAATTATAACTCCTGGGCCCTGCCCCACCCCTACTAAATCACAATTTCAGGAGGAGGGAC          
700

CTTCATTTTAACACTCACCCAGGTGATTTTTATGCTCCGAGGAGGTCCAGGGACTCCAAGTTAAGTACGGTACTGCTGTCTTATTCTTTATTCTAAATTT          
800

TAAGGTCTGCACAAATTGGTTGAACTAATGAGAAGAAAATTCAGCTTTAAAGCAGAAACACAGGTAGACGGTTGACAGAGTTCATCAAATGGATAATTGA          
900

AAATGTCCTCTGGACCCTAGCCATATAAGTTCTCTTCAAGGGTCTTGGCTACAGGCAAATGAGAACCCGAAAGGCTATTTGCTCTTTTGCTGCGGGCAGT          
1000

GGTGGGGGTGGAGGGCGGGGGAGGATTAACTGAGCCAGTTCTGCCCCCACCCTCGAATCACCTACCCCCACTCTGGTTAAAGCAGAAGACTTTTTATTTA          
1100

                                                                 
TAL1beta/ITF2-

                                                        
          FOXO4+          

TCTTGGCTGCCCTGGTTCGTTATTAAAAGGGTTAGCTTATACGTGTGTTTGCTGGGGCTGGAAGTGAAAACATCTGCAAAAGCATGCAATGCCCTGGAAC          
1200

                 
GATA1-   BRN2+/-       
GFI1-         
S8-                  
MEF2-          GATA1-

GGAACTCTTCTAATAAAAGATGTATCATTTTAAATGCGCTGAATTTTGATTCTGGTAATTCGTGCACTAGAGTGTCTATTTCGAGGCAGCGGAGGTATCA          
1300

                                 
MYCMAX-

         
CREB/ATF+               
USF-       EGR2-   
IK1-                               
MAZ-

TATGACAGCGCACGTCAAGGCACCGTGGAGCCCTCTCGTGGACTCCCACCCACTTTCCCATTCACCGCGGAGAGGGCTGCTCTCGCTGCCGCTCCCCCCG          
1400

                                                                                            
GATA- 

                                                        
SREBP1-              
   OCT1+/-    BRN2+ 

GCGAACTAGCATGAAATCTCCCTGCCTCTGCCGAGATCAAATGGAGCTTCTCGCTGATGGGGTGCGAGTATTACCTCCGCCATGCAATTTCCACTATCAA          
exon IV

  
S8                                                                                              

TAATTTAACTTCTTTGCTGCAGAACAGAAGGAGTACATACCGGGCACCAAAGACTCGCGCCCCCTCCCCCCTTTAATTAAGCGAAGGGAACGTGAAAAAA          
1600

TAATAGAGTGTGGGAGTTTTGGGGCCGAAGTCTTTCCCGGAGCAGCTGCCTTGATGGTTACTTTGACAAGTAGTGACTGAAAAGGTGGGTTTGTTTTCTT          
1700

TCTTTCTCTTTCCGTTTTTCTGTTTGGTCGGCTAGAAAGCGTGTGGCTTTAGCGAGGTCTGTCATTGCCTGGGCTTCCTGGCTGGAACAAGTAACTTGGT          
1800

                           
MMEF2+            
GFI1-

           
KROX-            
BRN2+      
GATA1+/-                                      

GTAACGTTATCTGGGGGCGTTCATCAATAAAAAATGCTGTTATTATCTTGATTGAATTCCTATTAGGCAAACTCTAGAGAGGTCAGTGCGCGAACTCTGT          
1900

TTAAGCCGGCGTGTTTAAGGCAGCAGAGTAAACCAATAGCCCCCATGCTCTGTGCGATTTCATTGTGTGCTCGCGTTCGCAAGCTCCGTAGTGCAGGAAG          
exon V

    IK1+                                 MAZ-       

GTGCGGGAAGGTGTGTCTGTGGCCCGGGAAACGCACGCCCTCTCCCAGAGAACTTGGGTGCTGGGATGGGGAGGAAGGGGAGAGTTGAAAGCTAGGGGAG          
2100

CGAGACCTCGGGGCGTGCGATTCTCACTCGCTCCCTCCCGCCCCAGCGCCCACAGCCGGGGTTTCTGCAGAGGGCGCGGGACGCGGGGTTCCCCGGGGCT          
exon Vh

GAGGCTGGGGCTGGAACACCCCTCGAAGCCGCGGGCGTCCTGTCCAAGGCGCCCCAGGAGGGCGCAGGACTCGCAGGGCGATGTCGCGGGGCCCTAGGGG    
     
2300

AGGAGGTGAGGACAGGCCCCGGGGGAGCGGGGAGTTCCGGGCGCCCCTCGGTTCCCCGCGCGAGGAAAAGACGCGGCGTTCCCTTTAAGCGGCCGCCTCG          
2400

                                                                     
GFI1+       SREBP+

AACGGGTATCGGTAGCGCGGGCGAGCGGGGAGCGGGGGGCGGGGGGCGGGGGGGGGGGGGCGGCGCCGTTTGACCAATCGAAGCTCAACCGAAGAGCTAA          
2500

ATAATGTCTGACCCGGGCGCAAGGCGCAGCCTGGAGCTCCGGGTCCCCGACGCTGCCGCCGCCGCGCCCGGGCGCACCCGCCCGCTCGCTGTCCCGCGCA    
     
2600

 

CCCCGTAGCGCCTCGGGCTCCCGGGCCGGACAGAGGAGCCAGCCCGGTGCGCCCCTCCACCTCCTGCTCGGGGGGCTTTAATGAGACACCCACCGCTGCT          
exon VI

                                                                                            
CREB+ 

GTGGGGCCGGCGGGGAGCAGCACCGCGACGGGGACCGGGGCTGGGCGCTGGAGCCAGAATCGGAACCACGATGTGACTCCGCCGCCGGGGACCCGTGAGG          
2800

TTTGTGTGGACCCCGAGGTAGGCAAGCGCTGGGAATGGGGCTTGGTGCAGGAGCTGCCCGTCCGCGGGAGAGAGTTGACTGGGGGATCCCCCACCCCAAA          
2900

GTTGTGGGACGAGGCCAGTCTCCTTCTTTCCTCCCCTCCGGTAGAAGGGACGATTTGGAGTTACTCTTGGGGAGTTTTCTCCCCCATCCCACAACCCAGA          
3000

AGGTCAGCCGGCACCACCAGGGAAAAAGGGACCCGGGGAAGTCACGAAGTAGAGGAGGGAAGGCCTGGAGGAGACCCAGAGCTGCGTGATGGGAGCAAAG          
3100

ACGGCGACCCGGGGATCCCTCGCAGCCCTCCCCCAGCCCAGGAGTAGTCGAGAGAGACTTAGGGGGCCAGAGCTGTCGAGGGTCCTGACTGAGGGGAGGG          
3200

TGCTGGGGCTAGGCTAGGAATCCTTCCAGGGGGTGGGTGGTCCCCGCGCCGACTTGCGGGGGGAGTGGGAGGGAAGCTTGCGCCTTCAGCCCGCATCCCT          
3300

TCCCCGGAGCTGCACACGGCTACCTGCTCCCCAGGAATTGAGACTGAAGTGGACTTACAAGTCCGAAGCCAATGTAGCTTGGAAAACTTGGGAGGCGGAA          
3400

TTCCTACCGCTGGGAACTGAAAGGGTCTGCGACACTCTCGGGCAGGCCGAACCCACATCTCTACCCATCCTGCGCCCCTCTTCTGAAGCGCCCTCCAGGG    
     exon VII

AAGTTAAGAGTTTTGACTTTCGGGGAGTGGTTGGGATGTACGTGGGGGATTCTTGACTCGGGTTAGTCTCTGGGGATGCAGAGCCGGGAAGAGGAATGG

 

 

 

 

 

 

BDNF
promoter and exon VIII

 

ATATTTTTTATAGAATTTTGACAGGGCCACTGTATAGGGGAAAGTCACTCCTCTTCCCCTTTATAGAAGAGTTGCACCTGGACAGTTGCATTGATGACTG          
100

TATCCAGTCTACACAAGAGGTCATTCCTGGGCATAAGAATGGACTGCCAAAATCTAGCTGAAACACCATTGACAAATAGACATTTTCTTTTGTTAATAAT          
200

ACCTGTGAAGGCTTTCATAACAGACATTTCCAGTTTTGTTCTCAGGCTCCTTGCAGCTGCTCCTCTAAAAGTGTGCTCTCTTCCAAGAGCTGACAATGGC          
300

CAGAAGCAAGGTGTTCTGTCTTTTGTGCCATCATCATCTAACTTGCCACACACATTTGGGATGTCAGCCTAGGTATAGGTTTTGTATCCACTCAGTATGG          
400

CTTGTGGGTCTGGTTGCCTTTGTTATTCATGCTGAGGGCCTCTGGGCATCAGTTTGGTGTGAGAGAACCCATTCCATGACCCTCCTTCCTTTGGCTGTTT          
500

TGACTCGATGGCTCTTGTTGGCACAGTCTGTGAGTGTCTGATGCTCTATCCATGCCGGACCATCTGTTCTGCTGTCTCTGTGGTCTGAAGTCGTTTTCTG          
600

AACTATTCCTTGATAATAAATTTGAGATGATCTTGTTCTACCTTTCTTTTCAAGTCACATCTTAGCCCCTTAGCCACATTCCCGAAGAACATGACAAATG          
700

GATGGGTCACAAGTCACGTAGCATAGGGTGTCAGACCACGAGGCTTTGAAGGGATTCTGTTGGGTGCTAAAAAGAAAGATTTTGTGTCACCACGATTTTT          
800

TTTAAAGGCATGTTGACACTTAGGCCTTAATTGAAAGCGTTCTTACTCAAGTAGAGTTGACAGAGGAGTATTTGGTAGTCGCGGTTGCTGGTCTGAAGAG          
900

CATGTGGTTCTGTTTCAATGCCCAATGAGATCTTCTCACGGGAAAATGTTCTGACATCTCAAACAAATGACCTTCATGCATAGTTTTGACAAAATACCCT          
1000

ATTAAGTATGCATATATGGTTGGTACCTTGTGGTAATAATTCAATACTGGAAACAGAGTAGCAACAAAGAAACATTAGGGTTATATTTAACCTCTGTGGA          
1100

ATTAGTGTGTAAACAAACTGCTTATCAGAAATGCTCATATGGGGCTTTGTTTAAATAAATAAGAAACTGGCATATAGGGTCTGCAGGATATTTCTGCCAA          
1200

GTAGACCTCCCTCACATTATAAGACACCACATCTATGTCTGACCCCATATGGAAAGAGGCATAGCAAGCCAGCACTGGTTCATATTCCCTCTCCACCACA          
1300

TAATGGGTATGTGATCTTAGGGAATCCACCGAAACTCTCTGGGCCTCAGTTTCCTCAGCTATAAATGGTGGATAATCAAATTATTTACCTCACCATTAAT          
1400

AAATGTTAGCTATTATTTTTTATCAAGTTTAATACAAAGAGAAACATTTTACTTATTTTTCCAGCTATCCAGAGCATCTTCCAAAATCCTATCACCAACA          
1500

AATACTGTATTGTATTTATTATAGCAACTATGTAAAAATGGAGTCCCTGTCCTATGCTTAGATGAAATATGTTGGTATTTGAGTTTGCATGTCTTCTATA          
1600

GGAATCAGTGTTTAGTGAAAACGGGTGGAGATAAACAGATGTTTTCACAGTCCTGTTGTTCACAGTACCGCCAAATTGAATGTTTCCATATAGGTGCATT          
1800

CTAATGGCTTAAATGATGCAGATATTTTCTGGCCAGCCATATGGATCTTTTGTCATCTAAGATGTTAATATTTTCCTTATATTTTATAGTAGTTCTGGAG          
1900

TACAGCCAGTTTCTTGAATAGGGTCCACATGGCTCATTATGCACAGGGCCTGGAAACTGCCTTACTCGTGCTGTTGAAATGAACCGTGACACTTCAGAAG          
2000

                                                                                   
ETS2-

AGCTGGGAGCTGGGGTAGAGCAGTGGCTAGGAGAACATATTCAATTATATTTCCTCCTGCATTAAGCTACAAGTAATGAGCACTTTCCTGTGCTTTACAG           2100

  
S8+/-                       
OCT1+

TTAAGTAATTAAAAGAAATTATAGAGTGGGATGCAAAAATAACCCGAAGGACAACTGGATGTGTGGAGCCACCAGTTTTCTCCATGAGTGCACAAGGTTA          
2200

 GATA1-                                                
FOXO4-                                 
    

ATCCTTGTTACTACTCAGAATGCTGAGTTTCTACAGAAAGGGTTGCAGGTCCACACATGTTTTGGCGTCTACCCACACGCTTCTGTATGGCATGACTGTG    
    exon VIII

CATCCCAGAAGAAGGGCTGTGCTGTGTACCTCCACGTTTCAGTGGAATTTAACAAACTGATCCCTGAAAATGGTTTCATAAAG

 

 

BDNF
promoter and exon IX

 

TGTGGTCACAAAATGAGGTGGTGAGGGCTGAGCCAAGATGGTGGCAGTGGGATGGATAAAAAGGGATGGCCAGGACAAATATTTTAAAGGAAAAATTAAC          
100

AGGACATCTTTACTGACTGGATTGGAAGGCTATGCAGGAAATATATTGTCAAACTTGATTCCAGGATTTCTATCCTATGCCTGGGTTGCCCAAAATATCA          
200

GGGAACCATTGTTAGAAAAGGTAGGAGATACCACTGTTCCAACAAAAAGTATTGAGTTTGGTGTTGCACCCACTTAACTTCAAGGCCTTACAAGTGAGTA          
300

GACAGTTAGTTAGAATTGCAGAAGTGCCACTCAGAGAGCAGGGCTTGCAATGTGGGGTTGGACTTTGTCACCATTGTGTTAATTCCTAATTCTATGCAGA          
400

TGCTCAGCTTGAGGAATACCCATGTTTGGGCTTCAGAATGAAAGCCAAGTAATATTTACTCAGATGCCAATTTTCCCTCTGAAATATTTGCTCATGGAAC          
500

TGAGAGAACAATATATAAAGCATTAATTATTTTTCTCATAAAGTTATTAATAAAAAGATAAGATCAGTGAAAGGCAGAGTAAACTAGAAGCCAAGTATAG          
600

AAAATGGTATCATTCAAAGACTCATTACTGTAGTGGTGAAAACAAAACAATTTTCCAACAGCTTAAGATGCCTCAGTATTTTGGACCATTTTTAAGTAGT          
700

TAGTGTGGGCACTTAGTAAATATGTATTAAACTATAGTTCATTAATTCTTTTTTTTTTTTTTTTGAGATGGAGTTTCACTCTGGTCACCCAGGCTGCATT          
800

TTTGCTTTCTTAGTGATATATAAAATGTCGAGTTTCACAATGATGGTATCTTAGATTTGATTAAATATGGTATTAAAAAATAGCTGATCACAGAAAGTCT          
900

CTACCAGTGTGATGTAGATGGCTAAAGTATTCCACATTTGCAAACTTTTATTGACCTAAATAAGAGGTGCCCCTTGGGTTGTTTTTATTTGGACTGGGAA          
1000

ATTAGGAGAAAGCTTTTTCATTCAGTGTGTAAGTACAATCTACCAGAAATAGAAACCCCCATGGACGATCTATTTCTTTGATGGTACAGGACTCAGAAC           
1100

ATTCACAAAGATTTAGTTGTTAGCGGAATAGACATCTGTATTTTATTCAAACCAATTTTCCCTTCCTAATCTGAGAACATTGTGCAATCTAAGCAGTTCT          
1200

AAGCATGTTTGCTATTCGTGCAAAGTGAGAGTAAATCTAAAAGAAATTTTTTTGTGTGTTTAGGGATGGTAATAAAGTCTCTTAGTGGTTGAAAATGTTA          
1300

TTTCTTACAAAAGTGGAGAACATTTGCTTTTCAATACCAGAGTTTTCAGCCATTTCTGCATTCTGACCTATTGACTGGAGGTAGGTTGCCTTTGAATTCA          
1400

GTAAAACTTCATGGGCAGAAACACAGTTCCTTTTCCTACTTATTTGGATATCATGATGGCCATTGCATGTATGTGTCTTTTTGTAAGTCCATGCCTCAGA          
1500

ACTGAGAAGTAGGAATAAAATTAGGGTCAGGGCTGGGGATGCTACTCTTTGCTGCTGAGAAACACAATGCTTCAGGTAAGTGATTCTGAAGTCCTTCACC          
1600

                                                            

ACCTGACGGTAACCTTGGGTTGGTCCATAGGTATGTTTTCATTTTGCTTGTTCATCCATTTTAATTGGCTTCCTAGAGCATGCTTGTAGATGTAGAGCCA          
1700

AATTTAGAGTAGAGCAACCCTCTGGCAAACAGGAAGAGATTAATTTTGTGGTATGCTTTTAAGGGACTTCCCAGGAAACTTCAAAAGCAGAAAAAGAAGC          
1800

ACTAGCTGCCTATTCCAAAATGTGTAAAACACCACTCAGCTTTTTAAAAGTAGGATAAACTCAGAGCGCGCGCACACGCGCGCGCGCACACACACACACA          
1900

CACACAGAGAGAACATCTCTAGTAAAAAGAAAAGTTGAGCTTTCTTAGCTAGATGTGTGTATTAGCCAGAAAAAGCCAAGGAGTGAAGGGTTTTAGAGAA          
2000

CTGGAGGAGATAAAGTGGAGTCTGCATATGGGAGGCATTTGAAATGGACTTAAATGTCTTTTTAATGCTGACTTTTTCAGTTTTCTCCTTACCAGACACA          
2100

TTGTTTTCATGACATTAGCCCCAGGCATAGACACATCATTAAAATGAACATGTCAAAAAATGATTTCTGTTTAGAAATAAGCAAAACATTTTCAGTTGTG          
2200

ACCACCCAGGTGTAGAATAAAGAACAGTGGAATTGGGAGCCCTGAGTTCTAACATAAACTTTCTTCATGACATAAGGCAAGTCTTCTATGGCCTTTGGTT          
2300

TCCTTACCTGTAAAACAGGATGGCTCAATGAAATTATCTTTCTTCTTTGCTATAATAGAGTATCTCTGTGGGAAGAGGAAAAAAAAAGTCAATTTAAAGG          
2400

CTCCTTATAGTTCCCCAACTGCTGTTTTATTGTGCTATTCATGCCTAGACATCACATAGCTAGAAAGGCCCATCAGACCCCTCAGGCCACTGCTGTTCCT          
2500

                                                                                           
FOXO4-     

GTCACACATTCCTGCAAAGGACCATGTTGCTAACTTGAAAAAAATTACTATTAATTACACTTGCAGTTGTTGCTTAGTAACATTTATGATTTTGTGTTTC    
  exon IXabcd

                             
S8+                                                                  

                           
MEF2+                
                                                  

                         
BRN2+/-                                                                  

TCGTGACAGCATGAGCAGAGATCATTAAAAATTAAACTTACAAAGCTGCTAAAGTGGGAAGAAGGAGAACTTGAAGCCACAATTTTTGCACTTGCTTAGA          
2700

AGCCATCTAATCTCAGGTTTATATGCTAGATCTTGGGGGAAACACTGCATGTCTCTGGTTTATATTAAACCACATACAGCACACTACTGACACTGATTTG          
2800

TGTCTGGTGCAGCTGGAGTTTATCACCAAGACATAAAAAAACCTTGACCCTGCAGAATGGCCTGGAATTACAATCAGATGGGCCACATGGCATCCCGGTG          
2900

                                      
                  MYOD+                                     
     

AAAGAAAGCCCTAACCAGTTTTCTGTCTTGTTTCTGCTTTCTCCCTACAGTTCCACCAGGTGAGAAGAGTGATGACCATCCTTTTCCTTACTATGGTTAT          
3000

                                                                          
MYOD+                    

TTCATACTTTGGTTGCATGAAGGCTGCCCCCATGAAAGAAGCAAACATCCGAGGACAAGGTGGCTTGGCCTACCCAGGTGTGCGGACCCATGGGACTCTG          
3100

GAGAGCGTGAATGGGCCCAAGGCAGGTTCAAGAGGCTTGACATCATTGGCTGACACTTTCGAACACGTGATAGAAGAGCTGTTGGATGAGGACCAGAAAG          
3200

TTCGGCCCAATGAAGAAAACAATAAGGACGCAGACTTGTACACGTCCAGGGTGATGCTCAGTAGTCAAGTGCCTTTGGAGCCTCCTCTTCTCTTTCTGCT          
3300

   MRF2+    
S8+              
FOXO4+                                                              

GGAGGAATACAAAAATTACCTAGATGCTGCAAACATGTCCATGAGGGTCCGGCGCCACTCTGACCCTGCCCGCCGAGGGGAGCTGAGCGTGTGTGACAGT          
3400

ATTAGTGAGTGGGTAACGGCAGCAGACAAAAAGACTGCAGTGGACATGTCGGGCGGGACGGTCACAGTCCTTGAAAAGGTCCCTGTATCAAAAGGCCAAC          
3500

TGAAGCAATACTTCTACGAGACCAAGTGCAATCCCATGGGTTACACAAAAGAAGGCTGCAGGGGCATAGACAAAAGGCATTGGAACTCCCAGTGCCGAAC          
3600

         
                    GATA1+                             
GATA1+                           

TACCCAGTCGTACGTGCGGGCCCTTACCATGGATAGCAAAAAGAGAATTGGCTGGCGATTCATAAGGATAGACACTTCTTGTGTATGTACATTGACCATT          
3700

  IK1+        

      GATA1+  

AAAAGGGGAAGATAG

 

 

 

 

 

 

BDNF
3'UTR

                                    

                                                
MEF2-                  
S8+ 

TGGATTTATGTTGTATAGATTAGATTATATTGAGACAAAAATTATCTATTTGTATATATACATAACAGGGTAAATTATTCAGTTAAGAAAAAAATAATTT          
100

                         
                             XFD2-

                                          
SOX5+    
MEF2-                        
FOXO4+

TATGAACTGCATGTATAAATGAAGTTTATACAGTACAGTGGTTCTACAATCTATTTATTGGACATGTCCATGACCAGAAGGGAAACAGTCATTTGCGCAC          
200

               
OCT1+   
                                         FOXO4+                     

AACTTAAAAAGTCTGCATTACATTCCTTGATAATGTTGTGGTTTGTTGCCGTTGCCAAGAACTGAAAACATAAAAAGTTAAAAAAAATAATAAATTGCAT          
300

       
SOX5-    GATA1+

        
S8-     
BRN2+                                
                               FOXO4+

GCTGCTTTAATTGTGAATTGATAATAAACTGTCCTCTTTCAGAAAACAGAAAAAAAACACACACACACACAACAAAAATTTGAACCAAAACATTCCGTTT          
400

                                                                                    
SOX5+

 
MMEF2-       
GATA1-                                        
GATA1+    S8+      
FOXO4+   OCT1-

ACATTTTAGACAGTAAGTATCTTCGTTCTTGTTAGTACTATATCTGTTTTACTGCTTTTAACTTCTGATAGCGTTGGAATTAAAACAATGTCAAGGTGCT          
500

GTTGTCATTGCTTTACTGGCTTAGGGGATGGGGGATGGGGGGTATATTTTTGTTTGTTTTGTGTTTTTTTTTCGTTTGTTTGTTTTGTTTTTTAGTTCCC          
600

           
IK1+                                                                      
TAL1beta/ITF2+

ACAGGGAGTAGAGATGGGGAAAGAATTCCTACAATATATATTCTGGCTGATAAAAGATACATTTGTATGTTGTGAAGATGTTTGCAATATCGATCAGATG          
700

        
MMEF2+

         
XFD2+

ACTAGAAAGTGAATAAAAATTAAGGCAACTGAACAAAAAAATGCTCACACTCCACATCCCGTGATGCACCTCCCAGGCCCCGCTCATTCTTTGGGCGTTG          
800

GTCAGAGTAAGCTGCTTTTGACGGAAGGACCTATGTTTGCTCAGAACACATTCTTTCCCCCCCTCCCCCTCTGGTCTCCTCTTTGTTTTGTTTTAAGGAA          
900

GAAAAATCAGTTGCGCGTTCTGAAATATTTTACCACTGCTGTGAACAAGTGAACACATTGTGTCACATCATGACACTCGTATAAGCATGGAGAACAGTGA          
1000

TTTTTTTTTAGAACAGAAAACAACAAAAAATAACCCCAAAATGAAGATTATTTTTTATGAGGAGTGAACATTTGGGTAAATCATGGCTAAGCTTAAAAAA          
1100

AACTCATGGTGAGGCTTAACAATGTCTTGTAAGCAAAAGGTAGAGCCCTGTATCAACCCAGAAACACCTAGATCAGAACAGGAATCCACATTGCCAGTGA          
1200

CATGAGACTGAACAGCCAAATGGAGGCTATGTGGAGTTGGCATTGCATTTACCGGCAGTGCGGGAGGAATTTCTGAGTGGCCATCCCAAGGTCTAGGTGG          
1300

AGGTGGGGCATGGTATTTGAGACATTCCAAAACGAAGGCCTCTGAAGGACCCTTCAGAGGTGGCTCTGGAATGACATGTGTCAAGCTGCTTGGACCTCGT          
1400

               
GATA1-                               

GCTTTAAGTGCCTACATTATCTAACTGTGCTCAAGAGGTTCTCGACTGGAGGACCACACTCAAGCCGACTTATGCCCACCATCCCACCTCTGGATAATTT          
1500

          
GATA1+                                                                 
FOXO4-

TGCATAAAATTGGATTAGCCTGGAGCAGGTTGGGAGCCAAATGTGGCATTTGTGATCATGAGATTGATGCAATGAGATAGAAGATGTTTGCTACCTGAAC          
1600

ACTTATTGCTTTGAAACTAGACTTGAGGAAACCAGGGTTTATCTTTTGAGAACTTTTGGTAAGGGAAAAGGGAACAGGAAAAGAAACCCCAAACTCAGGC          
1700

CGAATGATCAAGGGGACCCATAGGAAATCTTGTCCAGAGACAAGACTTCGGGAAGGTGTCTGGACATTCAGAACACCAAGACTTGAAGGTGCCTTGCTCA          
1800

ATGGAAGAGGCCAGGACAGAGCTGACAAAATTTTGCTCCCCAGTGAAGGCCACAGCAACCTTCTGCCCATCCTGTCTGTTCATGGAGAGGGTCCCTGCCT          
1900

CACCTCTGCCATTTTGGGTTAGGAGAAGTCAAGTTGGGAGCCTGAAATAGTGGTTCTTGGAAAAATGGATCCCCAGTGAAAACTAGAGCTCTAAGCCCAT          
2000

TCAGCCCATTTCACACCTGAAAATGTTAGTGATCACCACTTGGACCAGCATCCTTAAGTATCAGAAAGCCCCAAGCAATTGCTGCATCTTAGTAGGGTGA          
2100

GGGATAAGCAAAAGAGGATGTTCACCATAACCCAGGAATGAAGATACCATCAGCAAAGAATTTCAATTTGTTCAGTCTTTCATTTAGAGCTAGTCTTTCA          
2200

CAGTACCATCTGAATACCTCTTTGAAAGAAGGAAGACTTTACGTAGTGTAGATTTGTTTTGTGTTGTTTGAAAATATTATCTTTGTAATTATTTTTAATA          
2300

                 
GATA1-    

TGTAAGGAATGCTTGGAATATCTGCTGTATGTCAACTTTATGCAGCTTCCTTTTGAGGGACAAATTTAAAACAAACAACCCCCCATCACAAACTTAAAGG          
2400

ATTGCAAGGGCCAGATCTGTTAAGTGGTTTCATAGGAGACACATCCAGCAATTGTGTGGTCAGTGGCTCTTTTACCCAATAAGATACATCACAGTCACAT          
2500

GCTTGATGGTTTATGTTGACCTAAGATTTATTTTGTTAAAATCTCTCTCTGTTGTGTTCGTTCTTGTTCTGTTTTGTTTTGTTTTTTAAAGTCTTGCTGT          
2600

                                                                        
OCT1-

                 
FOXO4-                     
MMEF2-        NFkB-    BRN2+

GGTCTCTTTGTGGCAGAAGTGTTTCATGCATGGCAGCAGGCCTGTTGCTTTTTTATGGCGATTCCCATTGAAAATGTAAGTAAATGTCTGTGGCCTTGTT          
2700

             
GATA1+   OCT1+

CTCTCTATGGTAAAGATATTATTCACCATGTAAAACAAAAAACAATATTTATTGTATTTTAGTATATTTATATAATTATGTTATTGAAAAAAATTGGCAT          
2800

                    
BRN2+  

                        
SOX5-

                          
MRF2+

TAAAACTTAACCGCATCAGAAGCCTATTGTAAATACAAGTTCTATTTAAGTGTACTAATTAACATATAATATATGTTTTAAATATAGAATTTTTAATGTT          
2900

                                                                       
ARNT+              
XFD2+

                                                                         
FOXO4-      BRN2+/-

TTTAAATATATTTTCAAAGTACATAAAATCTGGGGGTTCTGGGTTTTTTTCTCTTTACTGTAAAACAAACATGAAAACGTGTTTTATTATAAATACGTGT          
3000

 

GTTCCTTGCTTTAAGACTAATCTGACTAGTTTTAGTAATTAAACAGCCCTCCAGAGAGGATACTTATTTGAAAGCCTGGGAGGCCAATCCTGCAGTCTGT          
3100

                                                                                            
GATA1+

GAGCTTCTGAGAAGTTGTAGGATTGGATCTGTCTTCCATGGTGGAAGATGGGAGTGTCATTTTCTCGGACACTTGAAAGTTTTACCCCTTTGAAGTGATA          
3200

TACTTTCATCAGATACCAAACCATGTATCCTAGCAACATCAGGATCCACGAATCACGGCTGGCTGGCTGGCTCTATTTGACTATAAAATAACTGTTAAAA          
3300

                      
GATA1+        
                                                              

AAAATCAAAGTAGGTTTACTTAGGGATAATGAGAATAAATTGGCCCAAATTTAGAAAGTCAAGTACTGGGGAAAAAAGTAAAAAAAGAAATTCAATATTT          
3400

                                       
OCT1-                 
GFI1-      
S8-       
              

GGAGACAGGTCACATATCCTCGGTTTGGAAGTGGTGCCATTTGCAGTATGTATTTGTATCCATAGCACTGATTTAATTTTTGTACGTGGAGCACACAGAT          
3500

  
GATA1-                                                                            
S8-          

TTTATATCATAGTACCCACAATCTAACAGATTGCTAGGATTTCTAAAAGGATTTGCCTCCGTGATGGCTGCCAAGCCGCTGGGTAATTTAGTTAAAGTGT          
3600

     
GATA1-         
IK1+            
BRN2+/-                            
TAL1beta/ITF2-            

TTAAAGTTATCACTAAATCTTAGGGAAATAAATGGAAGGATTTTAAATCAGAGAAGGGGCCATTAAGGAAAAAAAGCATCTGTTCTTACTCTTCAGCCTC                                                                                 
                                                                                        
3700

                                          
GATA1-    GATA1-     MEF2-    
FOXO4+                   

CCCATTTGAACACTGACAACCCATCCCATAACGTGGTGAACTATATCTGTGCAATGTATCATTTCTATTTCTAAACATACACACATGGTTTCCTCTTGTA          
3800

        
XFD2       
              FOXO4+                                           

TTGTCTTATAAATAAGGATCAGGAAAGTCATAACAAACATTGACTTGTGTTGTGTATATGGAAAAACAGATCGAATGAGATGAAT
